# Polyamine biosynthesis and eIF5A hypusination are modulated by the DNA tumor virus KSHV and promote KSHV viral infection

**DOI:** 10.1371/journal.ppat.1010503

**Published:** 2022-04-29

**Authors:** Guillaume N. Fiches, Zhenyu Wu, Dawei Zhou, Ayan Biswas, Tai-Wei Li, Weili Kong, Maxime Jean, Netty G. Santoso, Jian Zhu

**Affiliations:** 1 Department of Pathology, Ohio State University College of Medicine, Columbus, Ohio, United States of America; 2 Department of Genetics, School of Medicine, University of Alabama at Birmingham, Birmingham, Alabama, United States of America; 3 Gladstone Institute of Virology and Immunology, University of California, San Francisco, California, United States of America; 4 Department of Neurology, University of Rochester Medical center, Rochester, New York, United States of America; Florida State University, UNITED STATES

## Abstract

Polyamines are critical metabolites involved in various cellular processes and often dysregulated in cancers. Kaposi’s sarcoma-associated Herpesvirus (KSHV), a defined human oncogenic virus, leads to profound alterations of host metabolic landscape to favor development of KSHV-associated malignancies. In our studies, we identified that polyamine biosynthesis and eIF5A hypusination are dynamically regulated by KSHV infection through modulation of key enzymes (ODC1 and DHPS) of these pathways. During KSHV latency, ODC1 and DHPS are upregulated along with increase of hypusinated eIF5A (hyp-eIF5A), while hyp-eIF5A is further induced along with reduction of ODC1 and intracellular polyamines during KSHV lytic reactivation. In return these metabolic pathways are required for both KSHV lytic reactivation and *de novo* infection. Further analysis unraveled that synthesis of critical KSHV latent and lytic proteins (LANA, RTA) depends on hypusinated-eIF5A. We also demonstrated that KSHV infection can be efficiently and specifically suppressed by inhibitors targeting these pathways. Collectively, our results illustrated that the dynamic and profound interaction of a DNA tumor virus (KSHV) with host polyamine biosynthesis and eIF5A hypusination pathways promote viral propagation, thus defining new therapeutic targets to treat KSHV-associated malignancies.

## Author summary

Understanding virus-host interactions is crucial to develop and improve therapies. Kaposi’s sarcoma associated Herpesvirus (KSHV) is a human gamma-herpesvirus which deeply modulates the host metabolism and is associated with various cancers of endothelial and lymphoid origin. Polyamines are critical metabolites often dysregulated in cancers. In this study we demonstrated KSHV dynamically modulates polyamine metabolism to favor eIF5A hypusination and translation of critical KSHV latent and lytic proteins (LANA, RTA). Consequently, we found KSHV lytic switch from latency and *de novo* infection were dependent on polyamines and hypusination and pharmacological inhibition efficiently and specifically restricted KSHV infection. Our study provides new insights into KSHV alteration of the host metabolism and describe new therapeutic targets to treat KSHV-associated malignancies.

## Introduction

Polyamines, namely putrescine, spermidine and spermine, are low-molecular weight metabolites that are ubiquitous in eukaryotic life. Present in cells at millimolar concentrations, these polycations effectively binds DNA, RNA, and phospholipids thanks to their positive charge at physiological conditions [1] and, in turn, regulate many cellular processes, such as transcription, translation, cell cycle, chromatin remodeling and autophagy [2–7]. Consequently, their metabolism is tightly regulated by multiple layers of feedback loops and interconversion mechanisms [8,9]. Ornithine decarboxylase 1 (ODC1) is the rate-limiting enzyme that modulates the initial step of the polyamine biosynthesis pathway by converting ornithine into putrescine. Putrescine is then converted into spermidine by the spermidine synthase (SRM) which is subsequently converted into spermine by the spermine synthase (SMS). S-adenosyl methionine decarboxylase (SAMDC) catalyzes the conversion by providing the aminopropyl donor (decarboxylated S-adenosyl-L-methionine, dcSAM) required for SRM and SMS enzymatic reactions. In addition, catabolic enzymes, such as spermidine-spermine N1-acetyltransferase (SSAT-1), polyamine oxidase (PAO), and spermine oxidase (SMOX), can acetylate or oxidize polyamines to mediate their export and/or convert back to previous stages.

However, the homeostatic balance of polyamines is often disrupted in cancers. Indeed, upregulated metabolism of polyamines has been observed in cancers in order to fulfill the increased needs associated with tumorigenesis and rapid growth of tumor cells (reviewed in [10,11]). For instance, polyamines, particularly spermidine, are required for the hypusination of the eukaryotic initiation factor 5A (eIF5A). Both of spermidine and hyp-eIF5A have been found upregulated in multiple types of cancers [12–15]. Hypusination is a unique post-translational modification, where the spermidine is added to eIF5A. It is only reported to occur on eIF5A so far [16,17], which enables the selective control of mRNA translation through a conserved biochemical mechanism targeting “hard-to-translate” region, such as polyproline stretches [18–20]. Consequently, eIF5A hypusination has emerged as a key regulator of the polyamines’ downstream cellular processes, such as autophagy [21,22].

Furthermore, investigation of polyamines’ role in regulating viral infections started nearly 50 years ago. Earlier reports suggested that polyamines benefit the viral packaging of large DNA viruses, such as herpes simplex virus (HSV-1) [23], Vaccinia virus (VACV) [24], and polioviruses [25]. Only very recently, it has been reported that polyamines are involved in the viral life cycle of RNA viruses to promote transcription, translation, and viral packaging, including Zika and Chikungunya viruses [26], Ebola virus [27,28], Rift valley fever and LaCrosse viruses [29,30]. In return, viruses often manipulate the polyamine pathway, illustrated by herpesviruses. Human cytomegalovirus (HCMV) is known to induce the expression of ODC1 [31], while ODC1 inhibition using its inhibitor DFMO limits viral replication of HCMV [32]. HSV-1 upregulates the expression of SAMDC [33], while Epstein-Barr virus (EBV) downregulates SSAT-1 [34].

In contrast, there is almost no knowledge regarding to the functional contribution of polyamines to Kaposi’s sarcoma associated Herpesvirus (KSHV), a relatively recently discovered herpesvirus. KSHV is a human γ-herpesvirus and the etiological agent leading to Kaposi’s sarcoma (KS) [35,36] and associated with two lymphoproliferative disorders, primary effusion lymphoma (PEL) [37] and Multicentric Castleman Disease (MCD) [38]. KSHV has the capacity to establish a life-long infection in the infected individual and persist primarily in infected B lymphocytes and endothelial cells in a quiescent state. During viral latency, only a limited subset of viral latent genes is expressed. Latent infection of KSHV supports the maintenance of viral episomes, and also modulates host cellular environment, including regulation of metabolic pathways, to promote cell proliferation [39–41]. Latent KSHV can be reactivated, and expression of viral lytic genes is turned on and new virions are produced during viral lytic cycle. Although KSHV remains latent in most infected cells, which plays a major role in viral tumorigenesis, lytic replication of KSHV is critical for viral dissemination and also contributes to tumor progression [42,43]. Hence, it is critical to understand how latent KSHV shapes the host cellular environment to accommodate and benefit its lytic reactivation. In the present study, we reported that polyamine metabolism and eIF5A hypusination are modulated by KSHV, which in return promotes KSHV viral infection.

## Results

### KSHV dynamically modulates intracellular polyamines

**Fig 1:**
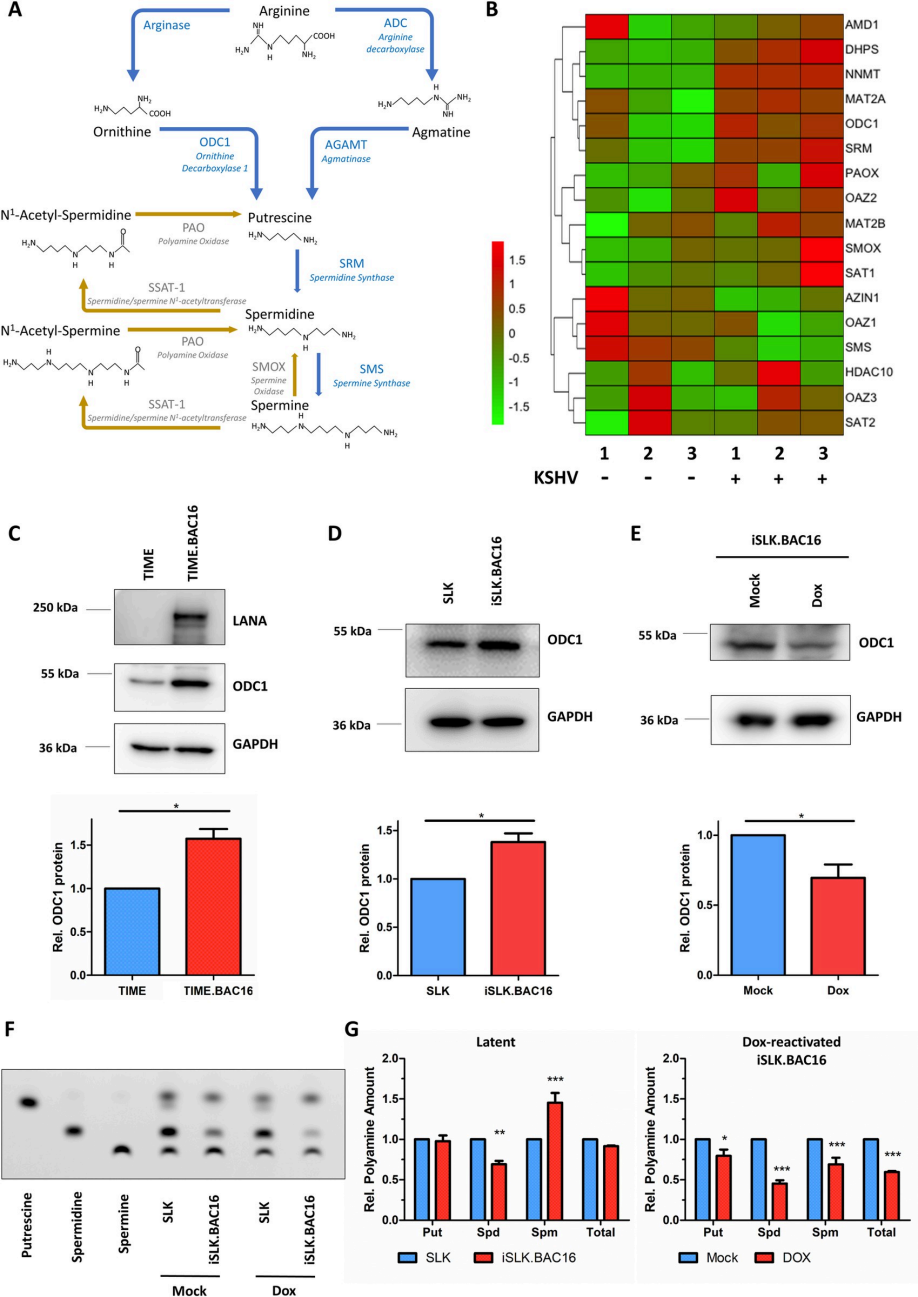
KSHV dynamically modulates the level of intracellular polyamines. (A). Schematic view of the polyamine biosynthesis pathway. (B). A heatmap with hierarchical clustering was generated to illustrate the dysregulation of polyamines pathway related gene expression in TIME cells latently infected with KSHV BAC16 in comparison to non-infected cells (GEO accession: GSE84237). (C-E). ODC1 protein in TIME cells latently infected with KSHV.BAC16 (8dpi) or uninfected TIME cells (C), or in KSHV latently infected iSLK.BAC16 cells or uninfected SLK cells (D), or in mock-treated or Dox-induced iSLK.BAC16 cells (E) was measured by protein immunoblotting. Intensity of protein bands was determined by using AlphaView SA (software) and normalized to GAPDH. (F, G). Intracellular polyamine species (putrescine [Put], spermidine [Spd], spermine [Spm]) in SLK and iSLK.BAC16 cells treated with Dox for 48h or mock were analyzed by thin-layer chromatography (TLC) with pure individual polyamine species used as reference (F). Relative changes of individual polyamine species (Put, Spd, Spm) or total three species in TLC results were determined, and normalized to latency (G, left panel) or dox-induced lytic reactivation (G, right panel). Results were calculated from n = 3–4 independent experiments and presented as mean ± SEM (* p<0.05; ** p<0.01; *** p<0.001, two-tailed paired Student t-test and 2-way ANOVA (Bonferroni test)).

We first analyzed the publicly available dataset (GSE84237) [44] to determine KSHV modulation of host genes relevant to polyamine metabolism **(Fig 1A)** in Tert-immortalized microvascular endothelial (TIME) cells latently infected with KSHV BAC16 strain. We observed multiple genes in polyamine pathway, including ODC1, SRM, SMS and DHPS, were significantly dysregulated due to KSHV latent infection **(Fig 1B)**. Similar analysis of a second publicly available RNA-seq dataset (GEO accession: GSE114625 [45]) further confirmed the upregulation of ODC1 and DHPS (**S1A Fig**) in BJAB cells latently infected with KSHV. Upregulation of ODC1 was further validated in TIME cells latently infected with KSHV BAC16 strain (TIME.BAC16) in comparison with its naïve TIME parental cells (**Fig 1C**). Such results were also observed in the KSHV latently infected cell line iSLK.BAC16 [46] in comparison with its naïve SLK parental cells (**Fig 1D**). Additionally, KSHV lytic reactivation in iSLK.BAC16 cells is induced by doxycycline (Dox) treatment since these cells are stably transduced with the Dox-inducible RTA (Replication and Transcription Activator) vector. Surprisingly, KSHV lytic reactivation resulted in the decrease of ODC1 protein in Dox-treated iSLK.BAC16 cells (**Fig 1E**).

Moreover, we analyzed the free, major polyamine species in these cells via thin-layer chromatography (TLC), including putrescine (put), spermidine (spd), and spermine (spm) (**Fig 1F and 1G**). We did not observe the dramatic difference of intracellular polyamines between iSLK.BAC16 and SLK cells without Dox treatment. We noticed the slight decrease of spermidine and increase of spermine, but overall polyamines remained the same (**Fig 1G, left panel**). Similarly, KSHV latent infection in TIME cells (TIME.BAC16) caused no significant change of polyamine species comparing to un-infected TIME cells (**S1B Fig).** We did observe the upregulation of ODC1 during KSHV latency, but regulation of intracellular polyamine homeostasis is multifaceted [11]. Cells can potentially compensate the ODC1 upregulation by exporting [47], catabolism [26] or consumption [16,17] of polyamines. For example, DHPS could use spermidine to generate hypusinated-eIF5A (hyp-eIF5A), and we indeed found that DHPS is upregulated due to KSHV latency in RNA-seq analysis (**Figs 1B and S1A**). On the contrary, all three polyamine species, particularly spermidine, significantly decreased in Dox-treated iSLK.BAC16 cells compared to no treatment (**Fig 1F and 1G, right panel**). This correlates with the reduction of ODC1 during KSHV lytic reactivation.

We also performed the above TLC assay in a KSHV-positive PEL cell line, TREx BCBL1-RTA, and BJAB cells (KSHV-negative B cell line). Similarly, we noticed that the polyamine levels were markedly decreased due to KSHV lytic reactivation in Dox-treated TREx BCBL1-RTA in comparison with TREx BCBL1-RTA cells without Dox treatment (**S1C and S1D Fig**). Consistently, we also confirmed that spermidine was the most reduced polyamine species in Dox-treated TREx BCBL1-RTA cells (**S1C and S1D Fig**). Taken together, these results clearly showed that KSHV infection distinctly and dynamically modulates the intracellular level of polyamines between latent and lytic phases.

### Polyamine synthesis enzymes are required for KSHV lytic reactivation

**Fig 2:**
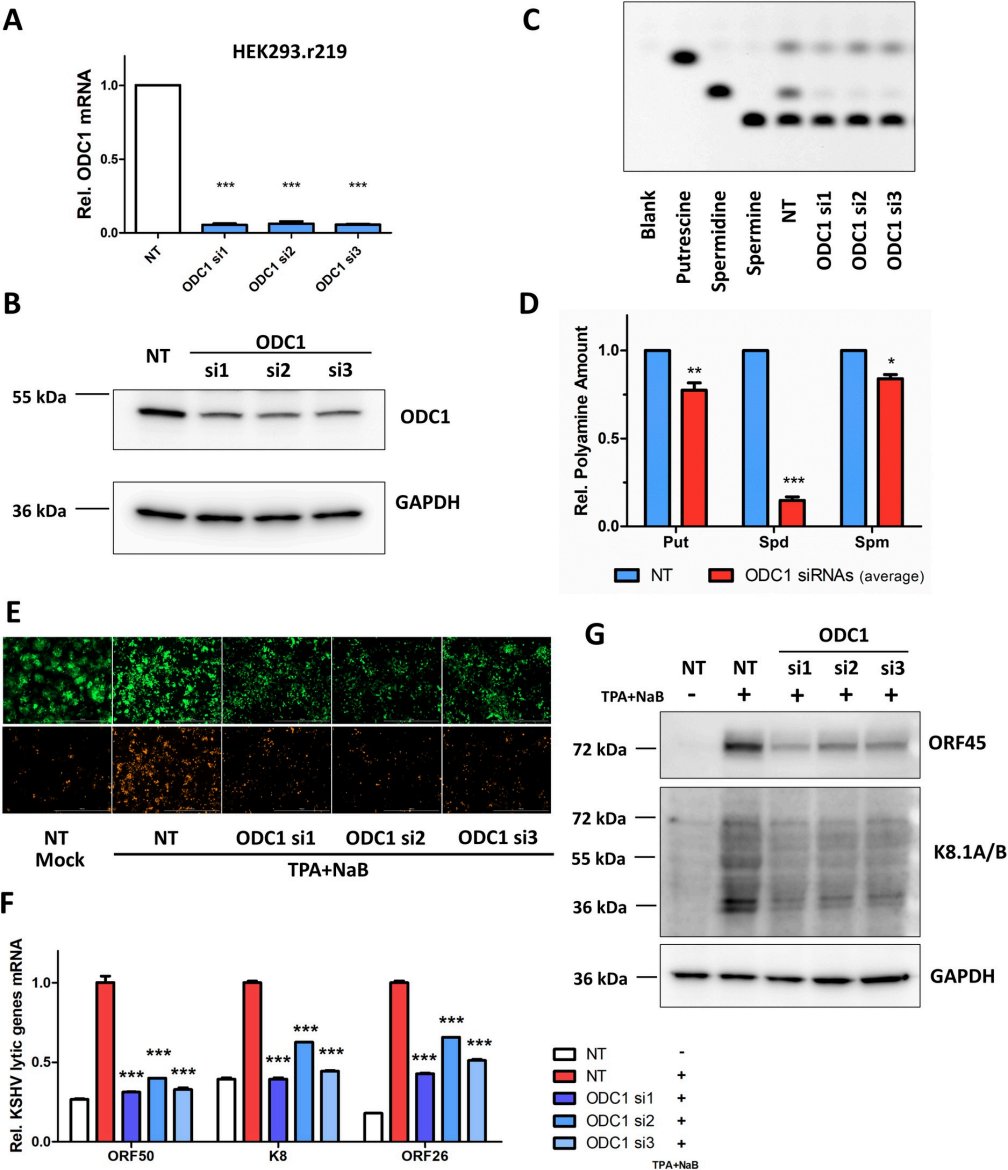
ODC1 is required for KSHV lytic reactivation. (A, B). ODC1 knockdown in HEK293.r219 cells transfected with ODC1 siRNAs (si1, si2, si3) or non-targeting control siRNA (NT) was analyzed by RT-qPCR (A) or immunoblotting (B). (C, D). Intracellular polyamine species (Put, Spd, Spm) in HEK293.r219 cells transfected with ODC1 siRNAs (si1, si2, si3) or NT were analyzed by TLC with pure individual polyamine species used as reference (C). Relative polyamine amount (Put, Spd, Spm) in above cells were quantified (average of ODC1 si1-3) and normalized to NT-transfected cells (D). Results were calculated from n = 2 independent repeats. (E-G). HEK293.r219 cells transfected with ODC1 siRNAs (si1, si2, si3) or NT were treated with TPA (20 ng/mL) + NaB (0.3mM) for 48h, and visualized by fluorescence imaging (E). Expression of RFP protein indicates KSHV lytic reactivation, while GFP signal means that cells are KSHV-infected. mRNA level of KSHV lytic genes (ORF50/RTA, K8/K-bZIP, ORF26) in above cells was analyzed by RT-qPCR assays and normalized to NT-transfected induced cells (F). Protein level of KSHV lytic genes (ORF45, K8.1A/B) was analyzed by immunoblotting assays (G). GAPDH was used as the loading control. Results were calculated from n = 3 independent experiments and presented as mean ± SEM (*** p<0.001, 1-way ANOVA (Tukey test) and 2-way ANOVA (Bonferroni test)).

We sought to further determine the contribution of the polyamine pathway to KSHV infection by investigating several key synthesis enzymes from this pathway. ODC1 is critical to catalyze the initial step of polyamine synthesis by converting ornithine into putrescine. HEK293 cells harboring a recombinant KSHV r219 strain (HEK293.r219) were transiently transfected with siRNAs targeting ODC1 or non-targeting (NT) control, and ODC1 knockdown was verified by qPCR and immunoblotting assays (**Fig 2A and 2B**). TLC assays showed that ODC1 knockdown led to the moderate reduction of putrescine and spermine, by 23% and 16% respectively, and the drastic depletion of spermidine by 85% reduction, averaged from three ODC1 siRNAs **(Fig 2C and 2D**), which also indicated that spermidine turnover is higher than for other polyamines in KSHV latently infected cells. KSHV r219 strain carries GFP and RFP fluorescent reporter genes allowing visualization of KSHV latent and lytic infection respectively [48]. KSHV lytic reactivation was induced by treating HEK293.r219 cells with 12-O-Tetradecanoylphorbol 13-acetate and Sodium Butyrate (TPA+NaB). Fluorescence imaging indicated that TPA+NaB-induced RFP expression was significantly reduced due to ODC1 knockdown for all three ODC1 siRNAs (**Fig 2E**). qPCR assays consistently showed that ODC1 knockdown by its three siRNAs significantly reduces the TPA+NaB-induced expression of KSHV lytic genes, including ORF50/RTA, K8/K-bZIP, and ORF26 genes, respectively representing immediate early, early and late lytic genes (**Fig 2F**), which is supported by the immunoblotting assays confirming the reduced protein level of KSHV lytic genes, ORF45 and K8.1A/B, in HEK293.r219 cells transfected with ODC1 siRNAs (**Fig 2G**). In addition, ODC1 knockdown even reduced the residual expression of KSHV lytic genes that occurs during spontaneous lytic replication (**S2A Fig**). The similar effect of ODC1 knockdown on KSHV lytic reactivation was observed in HEK293.r219 cells, in which KSHV lytic reactivation was alternatively induced by ectopic expression of KSHV ORF50 cDNA (**S2B and S2C Fig**), as well as in iSLK.BAC16 cells treated with Dox+NaB to induce reactivation of latent KSHV (**S2D and S2E Fig**). Taken together, data demonstrated the presence of ODC1 is required for the efficient KSHV lytic reactivation.

**Fig 3:**
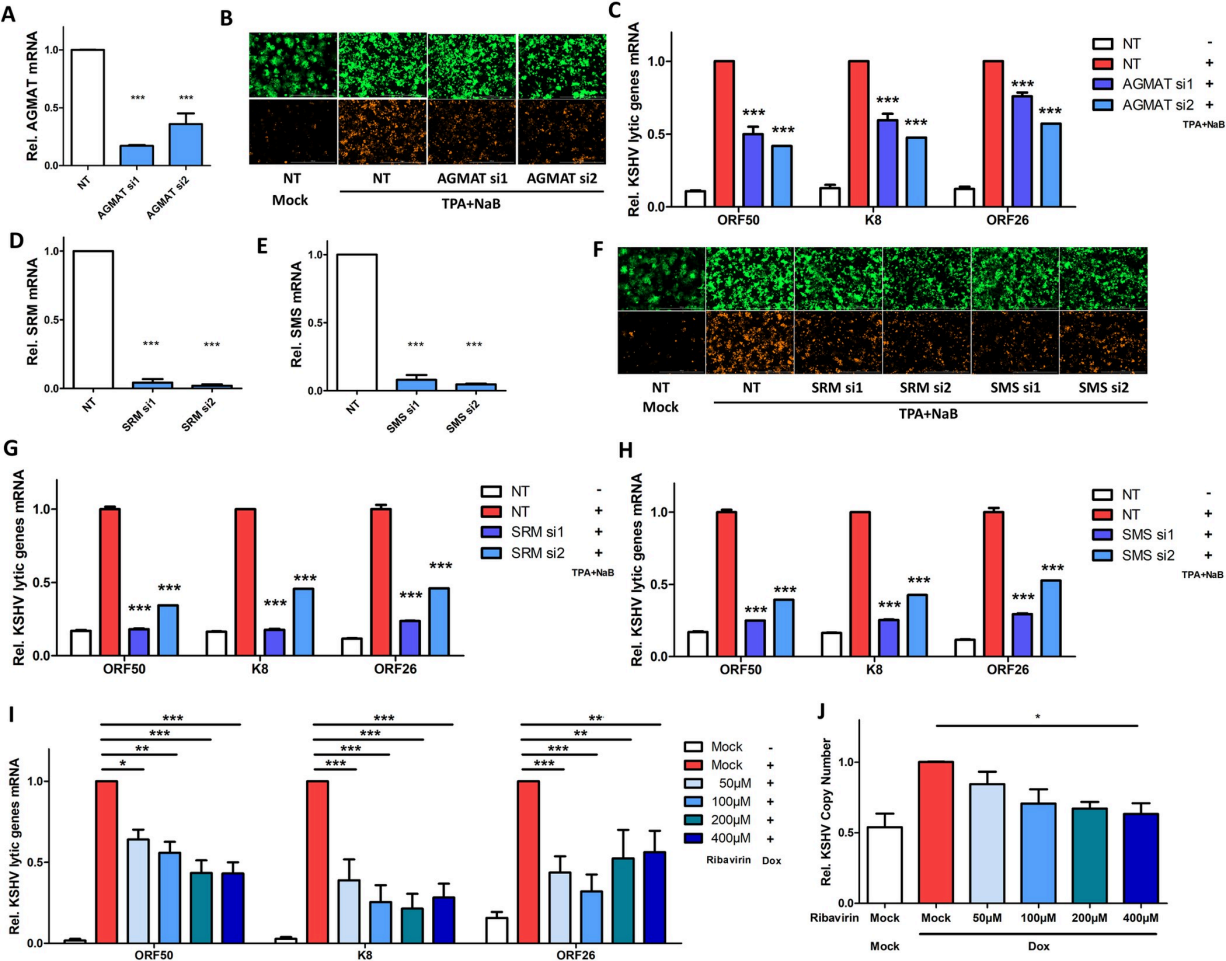
Other polyamine enzymes contribute to KSHV lytic reactivation. (A-C) AGMAT knockdown in HEK293.r219 cells transfected with AGMAT siRNAs (si1, si2) or NT was analyzed by RT-qPCR assays (A). HEK293.r219 cells transfected with AGMAT siRNAs (si1, si2) or NT was treated with TPA (20 ng/mL) + NaB (0.3mM) for 48h, and visualized by fluorescence imaging (B). mRNA level of KSHV lytic genes (ORF50/RTA, K8/K-bZIP, ORF26) in above cells was analyzed by RT-qPCR assays and normalized to NT-transfected induced cells (C). (D, E). SRM (D) or SMS (E) knockdown in HEK293.r219 cells transfected with their siRNAs (si1, si2) or NT was analyzed by RT-qPCR assays. (F-H). HEK293.r219 cells transfected with SRM/SMS siRNAs (si1, si2) or NT was treated with TPA (20 ng/mL) + NaB (0.3mM) for 48h, and visualized by fluorescence imaging (F). mRNA level of KSHV lytic genes (ORF50/RTA, K8/K-bZIP, ORF26) in SRM (G) or SMS (H) depleted, TPA+NaB-induced HEK293.r219 cells was analyzed by RT-qPCR assays and normalized to NT-transfected induced cells. (I, J). mRNA level of KSHV lytic genes (ORF50/RTA, K8/K-bZIP, ORF26) in iSLK.BAC16 cells pretreated with increasing doses of ribavirin for 24h and subsequently induced with Dox (1μg/mL, 48h) was analyzed by RT-qPCR assays (I). Copy number of KSHV genomes in above cells was also analyzed (J). Results were calculated from n = 3 independent experiments and presented as mean ± SEM (* p<0.05; ** p<0.01; *** p<0.001, 1-way ANOVA (Tukey test) and 2-way ANOVA (Bonferroni test)).

Using the similar approaches, we also determined the relevance of other polyamine synthesis enzymes to KSHV lytic reactivation. Beside ODC1, agmatinase (AGMAT) can convert agmatine to putrescine as well (**Fig 1A**). Although its importance in the polyamine metabolism was initially described for the lower organisms [49], AGMAT has only been recently studied for mammals [50]. Expression of AGMAT was significantly reduced by its two siRNAs in HEK293.r219 (**Fig 3A**), which caused the reduction of TPA+NaB-induced expression of RFP (**Fig 3B**) and KSHV lytic genes (**Fig 3C**) measured by fluorescence imaging and qPCR assays respectively. Putrescine is the shortest biogenic polyamine and converted to spermidine by the spermidine synthase (SRM), which is subsequently converted to spermine by the spermine synthase (SMS) (**Fig 1A**). Two siRNAs targeting SRM or SMS successfully led to their knockdown (**Fig 3D and 3E**), which also caused the reduction of TPA+NaB-induced expression of RFP **(Fig 3F)** and KSHV lytic genes **(Fig 3G and 3H)** measured by fluorescence imaging and qPCR assays respectively. SRM or SMS knockdown also reduced the residual expression of KSHV lytic genes that occurs during spontaneous lytic replication (**S3A and S3B Fig**). In addition, spermidine-spermine N1-acetyltransferase (SSAT-1) is a key catabolic enzyme in the polyamine pathway that acetylates spermidine and spermine, resulting in either their export out of the cell or their conversion by polyamine oxidase (PAO) to putrescine and spermidine respectively. Ribavirin is a FDA-approved drug that has been recently shown to induce SSAT-1 and thus deplete intracellular polyamines [51]. Our results identified that ribavirin severely impairs the Dox-induced KSHV lytic gene expression (**Fig 3I)** and viral genome amplification **(Fig 3J**) in iSLK.BAC16 cells in a dose-dependent manner which was sufficient to increase SSAT-1 protein level (**S3C Fig**) with limited cytotoxicity (**S3D Fig**) in these cells. Collectively, our investigation of several key polyamine synthesis enzymes verified that polyamine homeostasis is critical to KSHV lytic reactivation.

### Inhibition of polyamine synthesis efficiently blocks KSHV lytic reactivation

**Fig 4:**
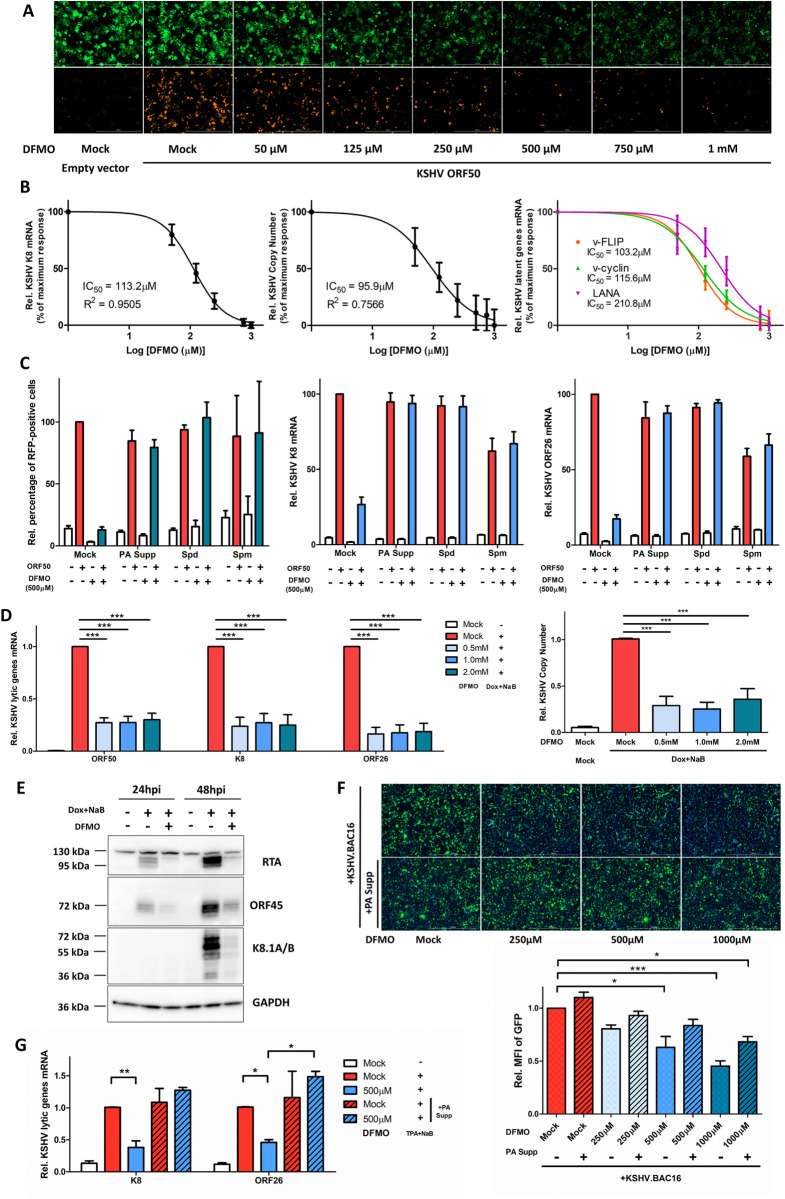
Polyamine depletion efficiently blocks KSHV lytic reactivation. (A, B). HEK293.r219 cells pretreated with progressively increasing doses of 2-difluoromethylornithine (DFMO) for 24h were subsequently induced by ectopic expression of ORF50 for 48h or left un-induced using the empty vector. Above cells were then visualized by fluorescence imaging (A). mRNA level of KSHV K8/K-bZIP (left panel) and latent genes (ORF71/v-FLIP, ORF72/v-Cyclin, and ORF73/LANA; right panel) in above cells were analyzed by RT-qPCR assays and normalized to mock-treated induced cells. The drug effect at a series of doses was plotted as percentage of maximum response by using GraphPad PRISM 5. Relative copy number of KSHV genomes (middle panel) was also analyzed. IC_50_ was determined for DFMO’s inhibitory effect. (C). Exogenous polyamines (mixed polyamines supplement [PA Supp, 5x], Spermidine [Spd, 10μM] or Spermine [Spm, 10μM]) were complemented into the culture media of HEK293.r219 cells treated with DFMO (500μM) and induced by ectopic expression of ORF50 for 48h or left un-induced using the empty vector. The above cells were labelled with Hoechst and visualized by fluorescence imaging to determine percentage of RFP-positive cells (left panel). mRNA level of KSHV lytic genes (K8/K-bZIP [middle panel], ORF26 [right panel]) in above cells were analyzed by RT-qPCR assays. All results were normalized to mock-treated ORF50-induced cells. (D, E). mRNA level of KSHV lytic genes (ORF50/RTA, K8/K-bZIP and ORF26) in iSLK.BAC16 cells pre-treated with increasing doses of DFMO for 24h and subsequently induced by Dox (1μg/mL) + NaB (1mM) for 48h were analyzed by RT-qPCR assays and normalized to mock-treated induced cells (D, left panel). Relative copy number of KSHV genomes in above cells was also analyzed (D, right panel). Protein level of KSHV lytic genes (ORF45, K8.1A/B) was analyzed by immunoblotting (E). GAPDH was used as the loading control. (F). TIME cells were *de novo* infected with KSHV BAC16 viruses (MOI = 1). Unbound viruses were washed away, and cells were subsequently treated with increasing doses of DFMO for 48h with or without addition of polyamines supplement (PA Supp, 5x) to the culture media. Cells were then subjected to fluorescence imaging analysis (top panel). Nuclei were labelled with Hoechst and used to normalized GFP fluorescence. MFI of GFP expression from nine different fields of view for ≥10^5^ cells was measured and normalized to mock-treated cells (bottom panel). (G). TIME.BAC16 cells were pre-treated with DFMO for 24h with or without addition of polyamines supplement (PA Supp, 5x) to the culture media. Cells were subsequently induced with TPA (20 ng/mL) + NaB (0.5mM) for 48h, and mRNA level of KSHV lytic genes (K8/K-bZIP and ORF26) were analyzed by RT-qPCR assays and normalized to mock-treated, induced cells. Results were calculated from n = 3–4 independent experiments and presented as mean ± SEM (* p<0.05; ** p<0.01; *** p<0.001, 1-way ANOVA (Tukey test) and 2-way ANOVA (Bonferroni test)).

Inhibitors have been developed to target specific enzymes or steps of polyamine pathway for treating various diseases, especially cancers. α-difluoromethylornithine (DFMO) is a well-tolerated, potent, and irreversible inhibitor of ODC1. Despite its poor pharmacokinetics, DFMO has significant therapeutic potential as a FDA-approved drug for treating trypanosoma (African sleeping sickness) [52], and it also has shown promising results in the on-going clinical trial for treating high-risk neuroblastoma, a severe form of pediatric tumor [ClinicalTrials.gov Identifier: NCT02679144] [53–55]. In our studies, DFMO was used as a chemical probe to inhibit ODC1 function and block downstream polyamine synthesis. Following 24hr treatment with DFMO, HEK293.r219 cells were transfected with an ectopic KSHV ORF50 cDNA to reactivate latent KSHV. DFMO reduced the RFP signal in a dose-dependent manner (**Fig 4A**). The qPCR assays of these cells also showed that DFMO significantly decreases expression of KSHV K8/bZIP early lytic gene and the copy number of KSHV genomes with comparable IC_50_ (drug concentration for 50% of maximal inhibitory effect) of 95.9μM and 103.2μM respectively (**Fig 4B**). DFMO also decreased the expression of KSHV latent genes (ORF71/v-FLIP, ORF72/v-Cyclin, and ORF73/LANA with IC_50_ = 103.2μM, 115.6μM, 210.8μM respectively). To confirm that DFMO’s anti-KSHV effect is through depletion of polyamines, we performed a rescue experiment by adding exogenous polyamines to the culture media of DFMO-treated (500μM ≈ IC_90_), ORF50-transfected HEK293.r219 cells. Supplementation with a polyamine mix (PA Supp), spermidine (Spd), or spermine (Spm) (**Fig 4C**) was able to restore the DMFO-inhibited lytic reactivation of KSHV through measurement of RFP-positive cells as well as expression of KSHV lytic genes (K8, ORF26) but caused no obvious cytotoxicity (**S4A Fig**). Likewise, DFMO inhibited the Dox+NaB-induced KSHV reactivation in iSLK.BAC16 cells as shown through qPCR analysis of copy number of KSHV genomes and KSHV lytic gene expression and immunoblottings (**Fig 4D and 4E**). Similar trend of DFMO was also observed in Dox-treated TREx BCBL1-RTA cells by immunoblottings of KSHV lytic proteins (**S4B Fig**). Alternatively, we also tested clofazimine (CLF), another FDA-approved drug that has been shown to inhibit ODC1 transcriptional activation [56]. CLF is used for treating drug-resistant tuberculosis [57] and has also demonstrated the potential for treating cancers, such as multiple myeloma [56]. Indeed, CLF treatment led to the reduction of Dox-induced KSHV lytic gene expression in iSLK.BAC16 cells in a dose-dependent manner without obvious cytotoxicity (**S4C and S4D Fig**). We further validated that polyamine biosynthesis is a specific target of DFMO for inhibiting KSHV infection in TIME cells. DFMO treatment severely impaired both KSHV *de novo* infection (**Figs 4F and S4E**) and TPA+NaB induced KSHV lytic reactivation in TIME cells (**Fig 4G**). More important, DMFO’s inhibitory effect was rescued by adding polyamine supplement to the cell culture media (**Fig 4F and 4G**). Overall, these results clearly demonstrated that polyamine synthesis is required for efficient KSHV lytic reactivation and *de novo* infection, and that FDA-approved drugs inhibiting the key enzyme ODC1 can be used to block KSHV lytic reactivation and the following viral dissemination.

### eIF5A hypusination is regulated by KSHV and required for its lytic reactivation

**Fig 5:**
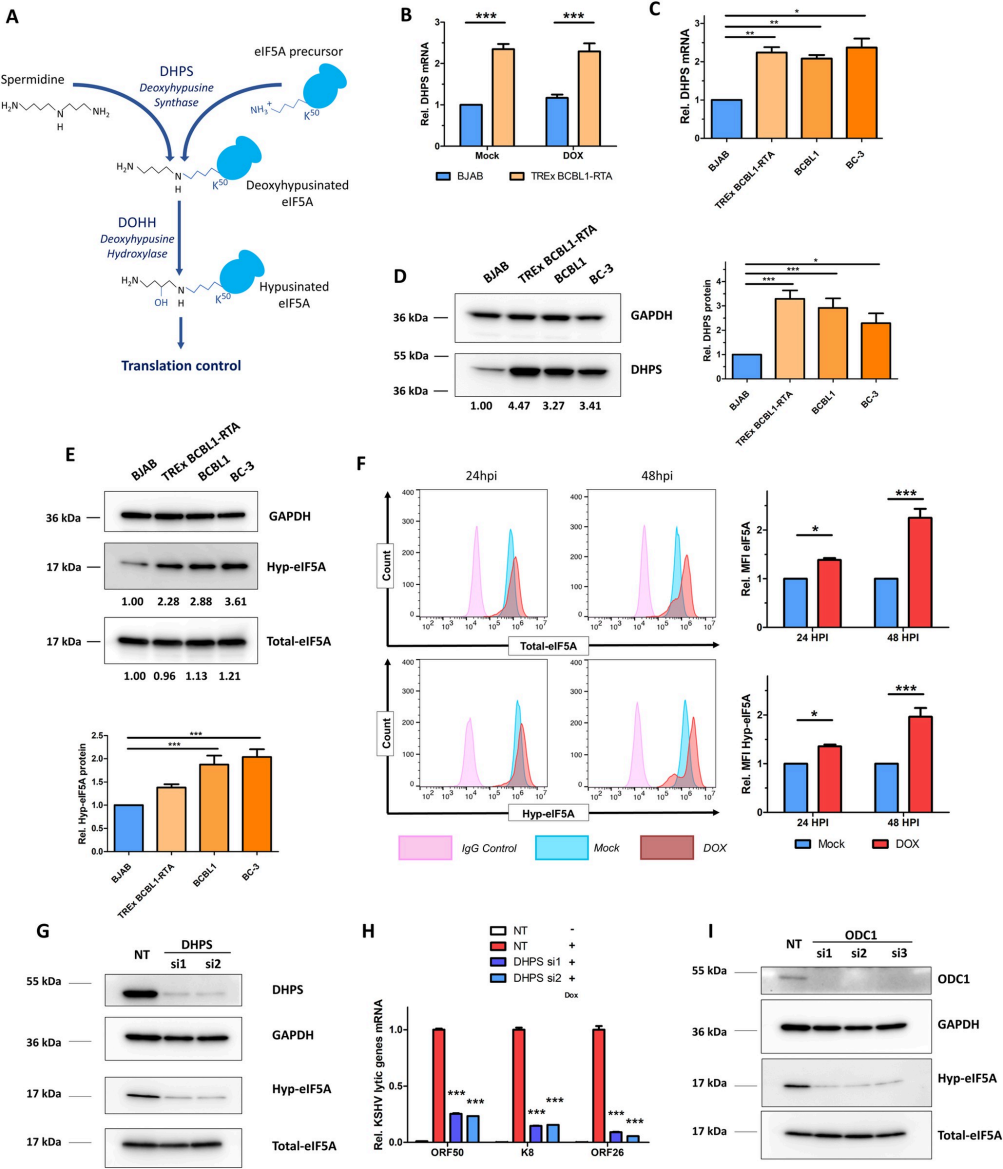
eIF5A hypusination is regulated by KSHV and required for its lytic reactivation. (A). Schematic view of spermidine consumption for eIF5A hypusination. (B). mRNA level of DHPS during KSHV lytic reactivation in TREx BCBL1-RTA (mock or Dox-treated, 48h) was analyzed by qPCR assay and compared to KSHV-negative BJAB cells under the same conditions. (C, D). mRNA (C) and protein (D) levels of DHPS in KSHV-infected PEL cells lines (BCBL1 [wild-type], TREx BCBL1-RTA, BC-3) and KSHV-negative BJAB cells were analyzed by qPCR and protein immunoblotting assays, respectively. (E). Protein level of total eIF5A and hyp-eIF5A in KSHV-infected PEL cells lines described in (D) was measured by protein immunoblotting. (F). Protein level of total eIF5A (top) or hyp-eIF5A (bottom) in TREx-BCBL1-RTA cells treated with Dox or mock were measured by dual-color immunostaining (Alexa fluor 488 for total eIF5A, and Alexa Fluor 647 for hyp-eIF5A) and flow cytometry at 24 and 48hpi. (G, H). DHPS knockdown and hyp-eIF5A protein level in iSLK.BAC16 cells transfected with DHPS siRNAs (si1, si2) or NT was analyzed by protein immunoblotting (G). mRNA level of KSHV lytic genes (ORF50/RTA, K8/K-bZIP and ORF26) in above cells induced with Dox (1μg/mL) for 48h was analyzed by RT-qPCR assays and normalized to NT-transfected, induced cells (H). (I). Effect of ODC1 knockdown on hyp-eIF5A protein level in iSLK.BAC16 cells transfected with ODC1 siRNAs (si1, si2, si3) or NT was analyzed by protein immunoblotting. Intensity of protein bands was determined by using AlphaView SA (software) and normalized to the loading control GAPDH. Results were calculated from n = 3–4 independent experiments and presented as mean ± SEM (* p<0.05; ** p<0.01; *** p<0.001, 1-way ANOVA (Tukey test) and 2-way ANOVA (Bonferroni test)).

Spermidine is the specific substrate of the deoxyhypusine synthase (DHPS) that catalyzes the formation of deoxyhypusinated eIF5A, followed by the deoxyhypusine hydrolase (DOHH) mediated hypusination of eIF5A at K50 [58] (**Fig 5A**). Such post-translational modification is unique and known to only occur to eIF5A [16,17]. Interestingly, DHPS gene expression increased in KSHV infected TIME and BJAB cells (**Figs 1B and S1A**). Thus, we postulated that spermidine-regulated eIF5A hypusination connects the upstream polyamine pathway to KSHV latency and lytic reactivation. We observed that DHPS mRNA is significantly upregulated in TREx BCBL1-RTA comparing to KSHV-negative BJAB cells (**Fig 5B**). However, KSHV lytic reactivation induced by Dox did not further increase DHPS mRNA (**Fig 5B**). In addition, KSHV latency-associated increase of DHPS gene expression was further validated in all three tested KSHV-positive lymphoma cell lines (TREx BCBL1-RTA, BCBL1, BC-3) at both mRNA and protein levels (**Fig 5C and 5D**). The protein level of hypusinated-eIF5A (hyp-eIF5A), but not total eIF5A, was also consistently higher in these KSHV-positive PEL cells (**Fig 5E**). The similar phenomenon was observed in iSLK.BAC16 cells comparing to SLK (**S5A Fig**). KSHV latently infected TIME (TIME.BAC16) cells also displayed the higher level of DHPS and hyp-eIF5A comparing to un-infected, parental TIME cells cultured in parallel (**S5B Fig**).

As we noticed that KSHV lytic reactivation triggers the increase of spermidine depletion (**Fig 1D**) without further upregulating DHPS gene expression (**Fig 5B**), we further examined the level of total and hyp-eIF5A protein during KSHV lytic reactivation. Dual-color immunostaining and flow cytometry analysis of Dox-induced TREx-BCBL1-RTA cells identified that there was a time-dependent increase of both total and hyp-eIF5A protein (**Fig 5F**). Increase of total and hyp-eIF5A protein was also observed in Dox-induced iSLK.BAC16 cells (**S5C Fig**) as well as in TPA+NaB-induced HEK293.r219 cells (**S5D Fig**). To confirm the key role of eIF5A hypusination in regulating KSHV lytic reactivation, we transfected siRNAs targeting DHPS or NT control in iSLK.BAC16 cells. DHPS was efficiently knocked down by its two siRNAs, which led to the significant decrease of hyp-eIF5A (**Fig 5G**) and a drastic inhibition of Dox-induced KSHV lytic gene expression in these cells (**Fig 5H**). We also confirmed that ODC1 knockdown by its siRNAs leads to the significant reduction of hyp-eIF5A without affecting its total protein level (**Fig 5I**). To summarize, our results demonstrate that eIF5A hypusination links the polyamine pathway to dynamic host-KSHV interaction and plays a critical role in regulating KSHV lytic replication.

### eIF5A hypusination determines the efficiency of KSHV protein synthesis

**Fig 6:**
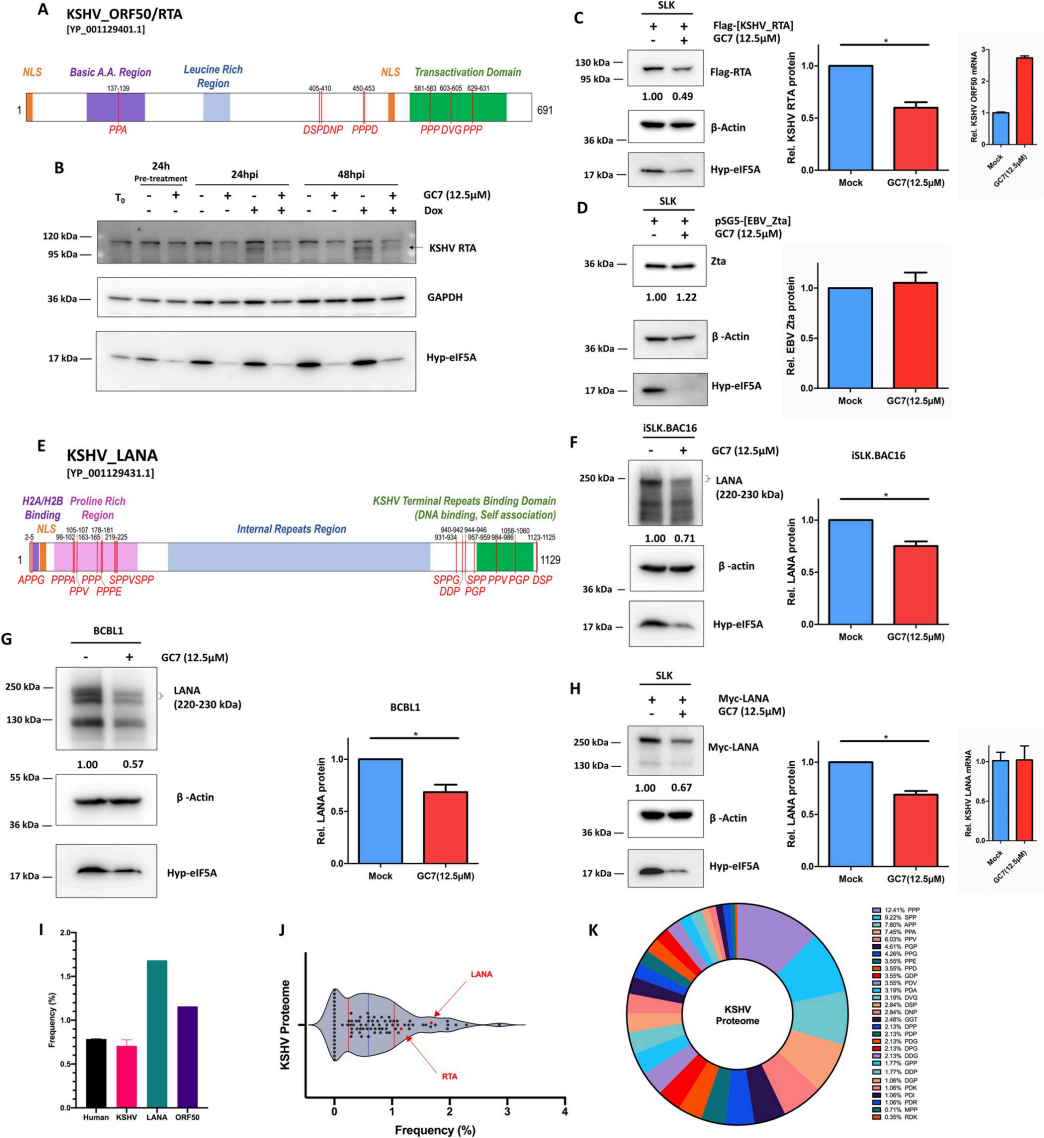
Translation of KSHV ORF50/RTA and LANA proteins requires eIF5A hypusination. (A). Schematic view of KSHV ORF50/RTA [YP_001129401.1] with hyp-eIF5A-dependency motifs annotated in red and several of its key domains. (B). Protein level of KSHV RTA, and hyp-eIF5A in iSLK.BAC16 cells pre-treated with GC7 (12.5μM) for 24h and subsequently induced with Dox (1μg/mL) up to 48h was analyzed by immunoblotting assays. GAPDH was used as the loading control. (C, D). Protein level of KSHV RTA (C) or EBV Zta (D) along with hyp-eIF5A in SLK cells transfected respectively with the pcDNA vector expressing Flag-tagged KSHV RTA or non-tagged EBV Zta and subsequently treated with GC7 (12.5μM) for 48h was analyzed by immunoblotting assays. β-actin was used as the loading control. mRNA level of transfected KSHV RTA in GC7-treated SLK cells was analyzed by RT-qPCR assays and normalized to mock-treated cells (C, right panel). (E). Schematic view of KSHV ORF73/LANA [YP_001129431.1] with hyp-eIF5A-dependency motifs annotated in red and several of its key domains. (F, G). Protein level of KSHV LANA along with hyp-eIF5A in iSLK.BAC16 (F) or BCBL1 (G) cells treated with GC7 (12.5μM) for 48h was analyzed by immunoblotting assays. β -actin was used as the loading control. (H). Protein level of KSHV LANA along with hyp-eIF5A in SLK cells transfected with the pcDNA vector expressing myc-tagged KSHV LANA and subsequently treated with GC7 (12.5μM) for 48h was analyzed by immunoblotting assays. β -actin was used as a loading control. mRNA level of transfected KSHV LANA in GC7-treated SLK cells was analyzed by RT-qPCR assays and normalized to mock-treated cells (H, right panel). (I). Frequency of hyp-eIF5A-dependency motifs in KSHV and human proteomes were analyzed and compared to that of KSHV RTA and LANA proteins. (J). Violin plot (quartiles in red, median in blue) illustrated the detailed distribution of the frequency of hyp-eIF5A-dependency motifs in KSHV proteome. Position of KSHV RTA and LANA proteins is highlighted. (K) Circular diagram showed the representation of the specific hyp-eIF5A-dependency motifs across the KSHV proteome. Intensity of KSHV protein bands was determined by using AlphaView SA (software) and normalized to β -actin. Results were calculated from on n = 2–3 independent experiments and presented as mean ± SEM (* p<0.05, two-tailed paired Student t-test).

Hyp-eIF5A plays a critical role in translation elongation by counteracting ribosome stalling during translation of difficult motifs, such as tri-proline (PPP) motif [20,59]. Schuller and colleagues recently described an extended repertoire of 29 motifs whose translation was dependent on hyp-eIF5A (**S6A Fig**) [18]. The polyamine-hypusine axis is activated in multiple types of cancers, in which protein synthesis is hypusine-addicted to support cell growth and metastasis, while inhibitors targeting the polyamine-hypusine axis can be used for anti-cancer therapies [12,14,60,61]. Since we also showed that KSHV infection modulates the polyamines-hypusine axis, we wondered whether eIF5A hypusination determines efficiency of KSHV proteins synthesis. We initially examined the protein sequence of KSHV ORF50/RTA [YP_001129401.1] for hyp-eIF5A-dependency motifs and identified 8 such motifs previously described by Schuller et al (**Figs 6A and S6F**). We tested the impact of eIF5A hypusination on KSHV ORF50/RTA mRNA translation by using the DHPS-targeted eIF5A hypusination inhibitor, GC7 (N1-guanyl-1,7-diaminoheptane) [22,62]. GC7 treatment led to the drastic reduction of Dox-induced KSHV RTA expression in iSLK.BAC16 cells (**Fig 6B**), which correlated with the reduction of hyp-eIF5A and caused neglectable cytotoxicity (**S6B Fig**). To further confirm, we ectopically expressed a cDNA of KSHV ORF50/RTA in SLK cells treated with GC7. Protein level of KSHV RTA was significantly lower due to GC7 treatment (**Fig 6C),** but its mRNA level was even moderately increased by GC7 (**Fig 6C, right panel**). On the contrary, we also tested GC7’s effect on expression of a GFP protein (CopGFP) that has no such difficult-to-translate motifs, which failed to lower protein level of GFP quantified by GFP-positive cells as well as MFI of GFP fluorescence (**S6C and S6D Fig**). Likewise, GC7 had no effect on EBV BZLF1/Zta protein level, since EBV BZLF1 only carries 2 hyp-eIF5A-dependency motifs (**Figs 6D and S6E**). Overall, these data confirmed that hyp-eIF5A is important and specifically controls KSHV RTA protein synthesis.

In parallel, we also examined the protein sequence of KSHV ORF73/LANA [YP_001129431.1] and identified 19 hyp-eIF5A-dependency motifs (**Fig 6E**), which are mostly conserved across multiple KSHV strains (**S6G Fig**). We tested the impact of GC7 on KSHV ORF73/LANA expression in KSHV latently infected cells, iSLK.BAC16 (**Fig 6F**) and BCBL1 (**Fig 6G**) cells. In both cases, GC7 led to the significant decrease of LANA protein. To further confirm, we ectopically expressed a cDNA of KSHV ORF73/LANA in SLK cells treated with GC7. Protein level of LANA was significantly lower due to GC7 treatment but not its mRNA level (**Fig 6H**). Therefore, our results showed that eIF5A hypusination is clearly required for synthesis of KSHV key lytic and latent proteins (RTA, LANA).

At last, we developed a bioinformatic program to efficiently identify the presence of hyp-eIF5A-dependency motifs, which was used to analyze both KSHV (S**1 Table**) and human proteomes. We recognized that the average frequency, as defined by the number of motif per amino acids of a protein, of the hyp-dependent motifs was similar between KSHV and human proteomes (respectively 0.71% and 0.78%; **Fig 6I and 6J**). KSHV RTA and LANA proteins both presented the much higher frequency (respectively 1.12% and 1.68%). We further examined the different representation of these motifs within the KSHV proteome (**Fig 6K**). For the list of 29 motifs, we observed the large discrepancy of representation for the specific motifs. For example, PPP accounted for over 12% of the hyp-dependent motifs, while other motifs, such as MPP, only accounted for 1% or less.

### Inhibition of eIF5A hypusination potently restricts KSHV viral infection

**Fig 7:**
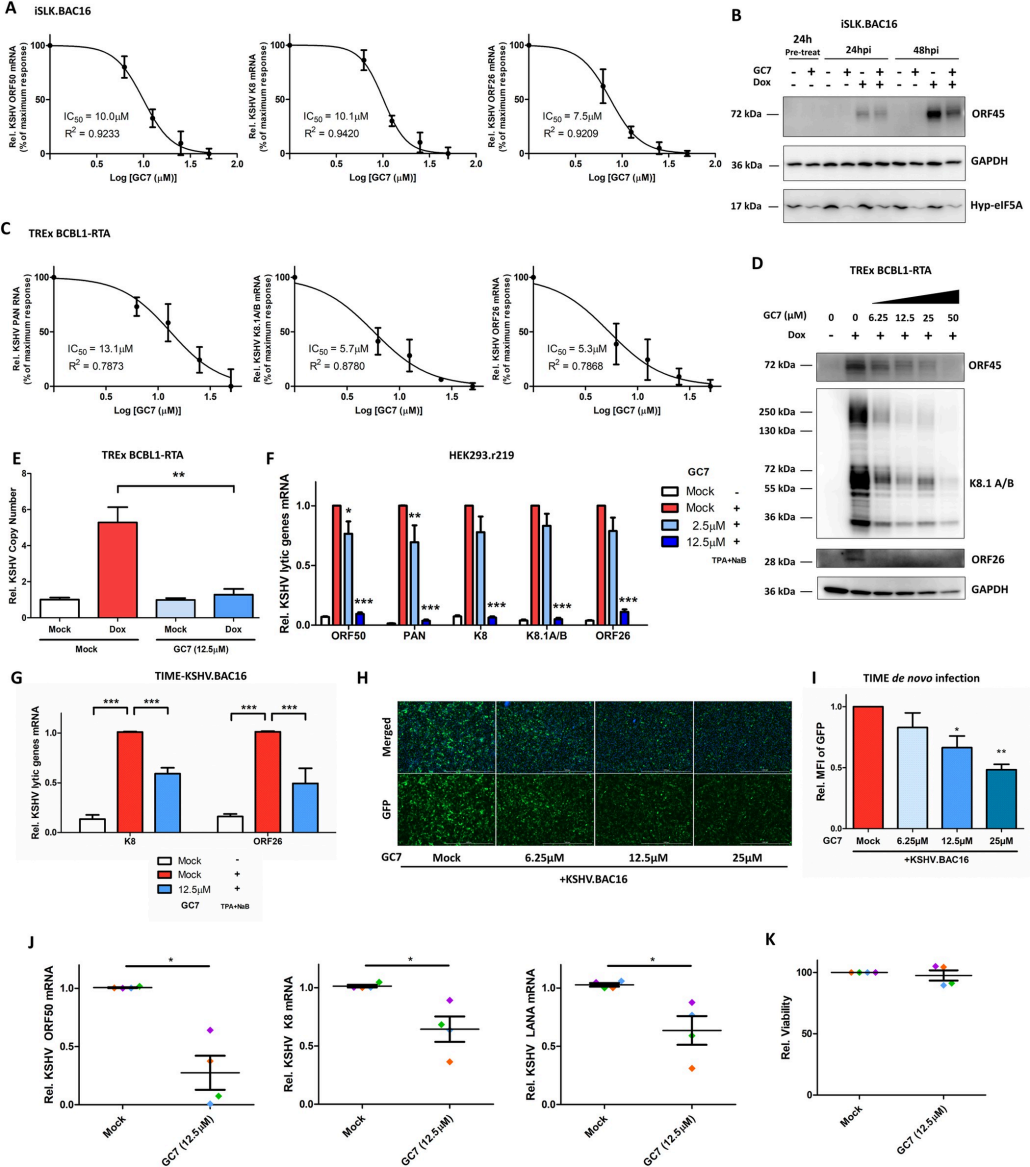
Inhibition of eIF5A hypusination efficiently blocks KSHV lytic infection. (A). mRNA level of KSHV lytic genes (ORF50/RTA, K8/K-bZIP and ORF26) in iSLK.BAC16 cells pre-treated with progressively increasing doses of GC7 for 24h and subsequently induced with Dox (1μg/mL) for 48h were analyzed by RT-qPCR assays and normalized to mock-treated induced cells. The drug effect at a series of doses was plotted as percentage of maximum response by using GraphPad PRISM 5. IC_50_ was determined for GC7’s inhibitory effect. (B). Protein level of KSHV ORF45 and hyp-eIF5A in iSLK.BAC16 cells pre-treated with GC7 (12.5μM) for 24h and subsequently induced with Dox (1μg/mL) up to 48h was analyzed by immunoblotting assays. GAPDH was used as the loading control. (C-E). mRNA level of KSHV lytic genes (PAN RNA, K8.1, ORF26) in TREx BCBL1-RTA cells pre-treated with progressively increasing doses of GC7 for 24h and subsequently induced with Dox (1μg/mL) for 48h were analyzed by RT-qPCR assays and normalized to mock-treated induced cells. The drug effect at a series of doses was plotted as percentage of maximum response by using GraphPad PRISM 5 (C). IC_50_ was determined for GC7’s inhibitory effect. Protein level of KSHV lytic genes (ORF45, K8.1A/B, ORF26) in above cells was analyzed by immunoblotting assays (D). GAPDH was used as the loading control. Relative copy number of KSHV viral genomes was also determined (E). (F). mRNA level of KSHV lytic genes (ORF50/RTA, PAN, K8, K8/K-bZIP, ORF26) in HEK293.r219 cells pre-treated with GC7 for 24h and subsequently induced with TPA (20 ng/mL) + NaB (0.3mM) for 48h was analyzed by RT-qPCR assays and normalized to mock-treated induced cells. (G). mRNA level of KSHV lytic genes (K8/K-bZIP and ORF26) in TIME.BAC16 cells pre-treated with GC7 for 24h and subsequently induced with TPA (20 ng/mL) + NaB (0.5mM) for 48h was analyzed by RT-qPCR assays and normalized to mock-treated induced cells. (H, I). TIME cells were *de novo* infected with KSHV BAC16 viruses (MOI = 1). Unbound viruses were washed away, and cells were subsequently treated with increasing doses of GC7 for 48h, then subjected to fluorescence imaging analysis (H). Nuclei were labelled with Hoechst (Blue), while GFP expression indicated infection with KSHV BAC16 viruses. MFI of GFP expression from nine different fields of view for ≥10^5^ cells was measured and normalized to mock-treated infected cells (I). (J, K). Primary tonsillar B cells isolated from 4 healthy donors were *de novo* infected with KSHV BAC16 viruses (MOI = 3). Unbound viruses were washed away, and cells were subsequently treated with GC7 (12.5 μM) for 72h, followed by RT-qPCR assays to measure mRNA level of KSHV lytic genes (ORF50/RTA, K8/K-bZIP) and latent gene (ORF73/LANA) and normalized to mock-treated cells (J). Cell viability was analyzed by CellTiter-Glo assays and normalized to mock-treated cells (K). Results were calculated from n = 3–4 independent experiments and presented as mean ± SEM (* p<0.05; *** p<0.001, two-tailed paired Student t-test, 1-way ANOVA (Tukey test) and 2-way ANOVA (Bonferroni test)).

Since we showed that synthesis of KSHV key lytic and latent proteins (RTA, LANA) is hypusine-dependent and can be efficiently blocked by GC7, we speculated that eIF5A hypusination would be a promising host target for developing anti-KSHV therapies. As a proof of principle, we tested the anti-KSHV potency of GC7 as an eIF5A hypusination inhibitor. Indeed, GC7 treatment suppressed the Dox-induced expression of KSHV lytic genes in a dose-dependent manner with similar IC_50_ = 10.0μM, 10.1μM, 7.5μM for ORF50/RTA, K8, ORF26 respectively (**Fig 7A**). It was confirmed by immunoblotting of KSHV ORF45 lytic protein in above cells (**Fig 7B**). Similarly, GC7 also suppressed KSHV lytic reactivation in Dox-treated TREx BCBL1-RTA cells in the dose-dependent manner (**Fig 7C and 7D**) without obvious cytotoxicity (**S7A Fig**). We also showed that GC7 severely impairs the amplification of KSHV viral genomes in these cells (**Fig 7E**). GC7 demonstrated comparable anti-KSHV effect in both HEK293.r219 and TIME.BAC16 cells treated with TPA+NaB to induce KSHV reactivation (**Figs 7F, S7B and S7G**). However, GC7 treatment failed to inhibit EBV lytic gene expression in Akata/BX cells treated with human IgG to induce EBV reactivation (**S7C Fig**). Overall, these results demonstrated that inhibition of eIF5A hypusination by GC7 potently and specifically restricts KSHV lytic reactivation but not EBV.

Furthermore, we determined the effect of GC7 on KSHV *de novo* infection. SLK cells were subjected to brief spin infection with KSHV.BAC16 viruses (MOI = 0.5). Unbound viruses were washed away, and cells were treated with GC7. KSHV infection rate was quantified by measuring the GFP expression from KSHV viral genomes in the infected cells. Fluorescence imaging showed that GC7 significantly reduces GFP expression in KSHV-infected SLK cells at 2 days post of infection (DPIs) in the dose-dependent manner (**S7D and S7E Fig**). Similarly, GC7’s inhibitory effect on KSHV *de novo* infection was demonstrated in TIME cells (**Fig 7H and 7I**), while GC7 showed no obvious cytotoxicity (**S7F Fig**). We also performed the similar assays using primary tonsillar B cells, which have been shown susceptibility to KSHV infection and support active lytic replication of KSHV for up to 5 DPIs [63]. Hence, primary tonsillar B cells from healthy donors were isolated and subjected to brief spin infection with KSHV.BAC16 viruses (MOI = 3). Unbound viruses were washed away, and cells were treated with GC7. Expression of KSHV lytic (ORF50/RTA, K8) and latent (ORF73/LANA) genes at 3DPIs were analyzed by qPCR assays, which clearly showed that GC7 significantly reduces KSHV viral gene expression in tonsillar B cells from four donors (**Figs 7J and S7G**) without any cytotoxicity (**Fig 7K**). These results suggested that eIF5A hypusination also participates in the early steps of viral life cycle during KSHV *de novo* infection and that its inhibition by GC7 potently blocks early events and prevents establishment of following persistent KSHV infection.

## Discussion

Our studies demonstrated that polyamine synthesis and eIF5A hypusination are dynamically regulated by KSHV infection. ODC1, the rate-limiting enzyme of the polyamine synthesis pathway, was found upregulated due to KSHV latent infection (**Fig 1**), consistently with the previous finding that ODC1 gene expression is subjected to transcriptional regulation by c-Myc [64,65] that is upregulated by LANA [66]. Overall, KSHV latency did not cause the drastic difference of total intracellular polyamines, verified in three types of cells harboring latent KSHV (iSLK.BAC16, TIME.BAC16, and TREx BCBL1-RTA). However, we did notice that spermine slightly increases while spermidine moderately decreases in iSLK.BAC16 cells without Dox treatment, perhaps due to the distinct polyamine compensation mechanism in this particular cell model. This also seems to correlate with increased levels of hyp-eIF5A in KSHV latently infected cells (**Fig 5**). Indeed, DHPS, the first key enzyme responsible for eIF5A hypusination, was significantly upregulated due to KSHV latency (**Fig 5**). Additionally, ODC1 knockdown resulted in a striking decrease of spermidine, much more dramatic than putrescine and spermine, which further indicate the higher turnover rate of spermidine (**Fig 2C and 2D**). High hyp-eIF5A benefit the synthesis of KSHV key latent protein ORF73/LANA, which is shown to depend on eIF5A hypusination in our own studies (**Fig 6**). On the contrary, KSHV lytic reactivation triggers an overall decrease of intracellular polyamines, particularly a drastic depletion of spermidine (**Fig 1**), correlating with the decrease of ODC1 expression during KSHV lytic reactivation (**Fig 1**) as well as the further increase of hyp-eIF5A in KSHV-reactivated cells (**Figs 5 and S5**). This is likely required to fulfill the even higher demand of KSHV viral protein synthesis during lytic reactivation, since the majority of KSHV genes (over 100) is expressed in lytic phase while only a dozen in latent phase. We indeed showed that eIF5A hypusination is critical to mRNA translation of KSHV key lytic protein ORF50/RTA (**Fig 6**). Protein synthesis of other KSHV lytic genes may also depend on hyp-eIF5A, which needs further investigation, as we identified that multiple KSHV viral proteins contain a high frequency of hyp-eIF5A-dependency motifs (**Fig 6K**). There could be also other explanations for KSHV reactivation induced spermidine drop. For instance, polyamines are strongly charged polycations, and it has been reported that spermidine and spermine are packed in HSV-1 virions [23], allegedly to neutralize the negative charge of its large viral genomes and help with DNA compaction. It still remains uncharacterized whether spermidine is also incorporated into KSHV virion as HSV-1.

In these studies, we also clearly showed that polyamine synthesis and eIF5A hypusination are critically required for KSHV infection. RNAi-mediated knockdown of several key enzymes participating in polyamine synthesis, including ODC1, AGMAT, SRM, and SMS, unanimously impairs KSHV lytic reactivation in multiple KSHV-infected cell systems (**Figs 2 and 3**), which is further supported by the results that several chemical probes inhibiting polyamine synthesis, including DFMO, clofazimine (CLF), and ribavirin, all block KSHV lytic reactivation in these cells (**Figs 3 and 4**). Furthermore, our studies also confirmed a correlation between eIF5A hypusination and the polyamine pathway, as we observed that ODC1’s RNAi-mediated knockdown and use of its inhibitor DFMO indeed represses eIF5A hypusination (**Fig 5**). In addition, a supportive finding is that knockdown of ODC1 seems to preferentially decrease spermidine that is the key polyamine species utilized for eIF5A hypusination (**Fig 2**). Likewise, we demonstrated that RNAi-mediated knockdown of the key enzyme DHPS participating in eIF5A hypusination as well as its inhibition by GC7 both efficiently suppress eIF5A hypusination and KSHV lytic reactivation in multiple KSHV-infected cell systems (**Figs 5 and 7**). We further unraveled that one potential mechanism is that synthesis of certain KSHV viral proteins could be hypusine-dependent (**Fig 6**), as activation of eIF5A depends on its hypusination by using spermidine [17], which enables eIF5A to alleviate ribosome stalling on defined hard-to-translate tri-peptide motifs [18,19]. We indeed recognized that KSHV ORF50/RTA and ORF73/LANA proteins carry multiple of such motifs that are conserved across all KSHV strains (**Figs 6 and S6**). Beyond KSHV lytic reactivation, inhibition of eIF5A hypusination by GC7 also impairs KSHV *de novo* infection, likely through the inhibition of both RTA and LANA protein translation [67] or by affecting synthesis of certain KSHV viral proteins possessing immune antagonizing or cell proliferating functions which are required for early steps of KSHV infection. For example, NF-κB is activated shortly post of KSHV *de novo* infection and important to the establishment of latency [68]. In addition, our studies validated the findings by using multiple cell models of KSHV infection, including epithelial (HEK293.r219, iSLK.BAC16), endothelial (TIME), and B lymphoma (TREx.BACBL1-RTA) cells, which unanimously demonstrated that polyamines and hyp-eIF5A are required for efficient KSHV lytic replication regardless of cell types.

It is interesting that both RTA and LANA may be targeted with the similar translational mechanism despite their canonically opposed functions. However, these proteins dynamics are primarily distinct as LANA and RTA critically determine the establishment of latent and lytic phases respectively. Therefore, the timing of their dependency on hyp-eIF5A through “hard-to-translate” motifs may be different, which would safely guard the initiation of lytic *vs* latent phase. In addition, although intranuclear LANA’s role in latency is well defined [69,70], several cytoplasmic LANA isoforms exist and have been linked to efficiency of KSHV lytic replication. For instance, cytoplasmic isoforms of LANA can (i) bind to cGAS and block STING-dependent ISG induction, interfering with cellular DNA sensing [71], (ii) interact with the MRN complex (Mre11-Rad50-NBS1) and antagonize NF-κB pathway activation [72]. Even though RTA and LANA majorly control lytic and latent phases respectively, their roles are important for both phases and can be intertwined at certain conditions. For instance, LANA can activate RTA at hypoxia [73], while RTA activation of LANA is critical to KSHV *de novo* infection [74]. Although KSHV might evolve to have both LANA and RTA commonly controlled by hyp-eIF5A-dependent protein translation, there are other mechanisms that further distinctly regulate their protein stability and functions, such as protein posttranslational modifications.

**Fig 8:**
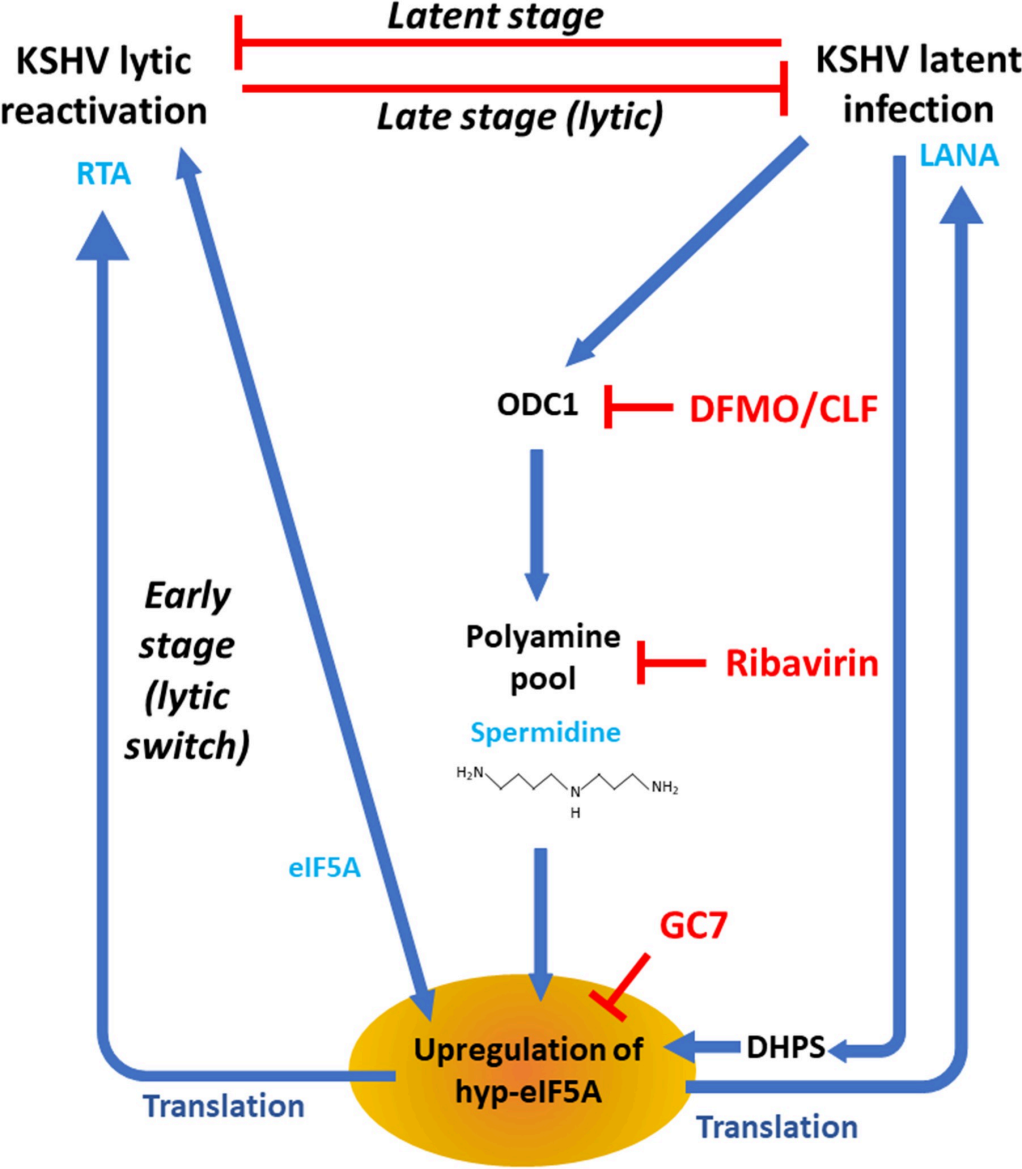
A proposed model illustrates the dynamic and profound interaction of host polyamine biosynthesis and eIF5A hypusination with KSHV infection. At the stage of latent infection, KSHV latent proteins transcriptionally activates ODC1, the rate-limiting enzyme of polyamine biosynthesis. However, spermidine is consumed to produce hypusine-eIF5A ensuring the efficient translation of LANA protein required for maintenance of KSHV latency. This is supported by that KSHV latency upregulates DHPS expression, leading to the increase of eIF5A hypusination and spermidine consumption. This results in a positive feedback sustaining activation of the polyamine-hypusine axis. At the early stage of lytic switch, KSHV ORF50/RTA gene starts to be actively transcribed upon certain stimuli, and the constant high level of hypusine-eIF5A ensures the efficient translation of RTA protein and overall promotes KSHV lytic reactivation. Lytic switch of KSHV also drive an early upregulation of eIF5A and the correlated increase of hyp-eIF5A. On the contrary, at the late stage of lytic replication expression of KSHV latent proteins diminishes, leading to decrease of ODC1 and reduction of intracellular polyamines. Given the critical roles of polyamine biosynthesis and eIF5A hypusination in KSHV infection, inhibitors targeting these metabolic pathways would serve as novel antiviral reagents to efficiently block both KSHV latent and lytic replications.

By combining our new results with earlier knowledge regarding to host and viral controls of KSHV infection, we propose the following model describing the comprehensive roles of polyamine pathway and eIF5A hypusination in regulating KSHV viral infection (**Fig 8**). During the latent phase of KSHV infection, latent proteins upregulates ODC1 expression while RTA transcription is suppressed [75]. ODC1 upregulation promotes intracellular polyamine biosynthesis but little changes are observed as spermidine is actively and steadily depleted which correlates with upregulation of DHPS and increased hyp-eIF5A. Accumulated hyp-eIF5A further promotes translation of LANA. At the early stage of lytic switch, RTA transcription is activated, and the newly transcribed RTA mRNAs can thus be efficiently translated thanks to the pre-existing higher level of hyp-eIF5A that could be further increased. However, at the late stage of lytic phase, RTA expression is strong enough to counteract LANA and other latent proteins, which leads to the reduction of ODC1. At this moment, KSHV lytic protein synthesis, especially RTA, is already less dependent on hyp-eIF5A, so decrease of intracellular polyamines may generate much less adverse effect on KSHV lytic protein synthesis. Additionally, it has been shown that c-Myc expression is transcriptionally downregulated by the KSHV lytic gene vIRF4 [76], which would also contribute to the decrease of ODC1. ODC1 reduction decreases intracellular polyamines, and thus hamper cell cycle progression [77], which has been shown to favor KSHV lytic reactivation [78]. In addition, accumulated spermine would convert to spermidine and putrescine through SMOX and PAOX, which further generates hydrogen peroxide and oxidative stress that have also been shown to promote KSHV lytic reactivation [79,80].

The evidence is emerging that KSHV modulates cellular metabolic landscape in order to fulfill its particular needs to promote viral propagation and oncogenesis [39,40]. A recent study [41] reported that broad modifications of host metabolism, including induction of arginine and proline metabolism, occur in KSHV-infected cells in 3D culture. Arginine is a precursor metabolite for polyamine synthesis (**Fig 1A**), and it can be sequentially converted to putrescine via ODC1 and AGMAT, both of which were identified as critical to KSHV lytic reactivation in our studies (**Figs 2 and 3**). Furthermore, these authors also reported that ornithine, the direct substrate of ODC1, is induced in KSHV-infected cells, which resonates with our findings. Overall, these observations along with ours illustrated that KSHV profoundly reprograms the metabolic pathways of host cells to not only promote viral latency and oncogenesis but also favor viral lytic reactivation and dissemination of KSHV viruses. However, it is still not fully clear how KSHV infection modulates polyamine pathway and eIF5A hypusination. We believe that regulation of ODC1 by KSHV latency might be just one of mechanisms. Some large DNA viruses encode functional genes related to the polyamine metabolism, such as *Paramecium bursaria* chlorella virus (PBCV-1) virus that encodes genes equivalent to most of the biosynthesis pathway [81–83] in its 331-kb long genome. More recently, it has been reported that the viral genome of bovine gammaherpesvirus BoVH-6 encodes a viral protein similar to ODC1 [84].

After all, our understanding of polyamine pathway and eIF5A hypusination in the context of KSHV infection will pave the way for development of new antiviral and antitumor strategies to treat KSHV-associated malignancy. As the proof of principle in our studies, inhibitors targeting polyamine pathway (DFMO, clofazimine, ribavirin) and eIF5A hypusination (GC7) can be used as antivirals to block latent and lytic replications as well as *de novo* infection of KSHV (**Fig 8**). As the matter of fact, three of them are already FDA-approved drugs currently being prescribed for treating other diseases. Thus, these drugs are safe for future evaluation of their potential to treat KSHV-associated malignancy in clinic. Beyond inhibition of KSHV latent infection, interruption of KSHV lytic infection is equally important to not only block generation of new KSHV virions and their dissemination but also reduce viral tumorigenesis since KSHV lytic infection contributes notably to tumors as well by generating angiogenesis and anti-apoptotic phenotypes (reviewed in [42,43]). In conclusion, polyamine pathway and eIF5A hypusination play a comprehensive role in regulating KSHV infections, which serve as promising new drug targets for treating KSHV infection and KSHV-associated malignancy.

## Materials and methods

*Cell culture*. HEK293.r219 [48] and iSLK.BAC16 [46] cells were respectively obtained from Dr. Prashant Desai (Johns Hopkins) and Dr. Shou-Jiang Gao (University of Pittsburgh) and cultured in DMEM supplemented with 10% FBS under selection (HEK293.r219: 5 μg/mL puromycin; iSLK.BAC16: 1.2 mg/mL Hygromycin B, 2 μg/mL puromycin, 250 μg/mL G418). TREx BCBL1-RTA [85] cells were obtained from Dr. Jae Jung (Cleveland Clinic) and maintained in RPMI supplemented with 10% FBS under selection (200 μg/mL Hygromycin B). TIME cells [86] were obtained from Dr. Shou-Jiang Gao (University of Pittsburgh) and maintained in Vascular Cell Basal medium complemented with Endothelial Cell growth kit (ATCC, Cat#PCS-100-030 and PCS-100-041). TIME.BAC16 cells were maintained identically up to 14 days post-infection, and checked for LANA and GFP protein expression. BC-3, BCBL1 and SLK cells were acquired from the NIH AIDS Reagent Program. These cells were maintained in RPMI supplemented with 10% FBS (BC-3 with 20% FBS). BJAB and Akata/BX cells were acquired from Dr. Renfeng Li (Virginia Commonwealth University) and maintained in RPMI supplemented with 10% FBS (Akata/BX under selection 500μg/mL G418). Tonsillar mononuclear cells and B lymphocytes were maintained in RPMI (Gibco) supplemented with 10% heat-inactivated human serum (Advanced Biotechnologies Inc, Cat#P2-203), 100 U/mL penicillin, 100 μg/mL streptomycin, 0.5 μg/mL Amphotericin B, and 50 μM β-Mercaptoethanol as previously described [87]. KSHV lytic reactivation was induced in TREx BCBL1-RTA cells with 1μg/mL doxycycline, in iSLK.BAC16 cells with either 1μg/mL doxycycline alone or in combination with 1mM Sodium Butyrate (NaB), in HEK293.r219 cells with 20ng/mL 12-O-Tetradecanoylphorbol 13-acetate (TPA) and 0.3mM NaB or ectopic expression of KSHV ORF50/RTA cDNA, and in TIME.BAC16 cells with 20ng/mL TPA and 0.5mM NaB.

### Compounds

12-O-Tetradecanoylphorbol 13-acetate (Cat#P8139) and sodium butyrate (Cat#AAA1107906) were purchased from Sigma-Aldrich and Fisher Scientific, respectively. Doxycycline was obtained from Fisher Scientific (Cat#BP2653-1). Polyamine supplement (1000x) (Cat#P8483), putrescine (Cat#P5780), spermidine (Cat#S0266), spermine (Cat#S4264), and clofazimine (Cat# C8895) were purchased from Sigma-Aldrich. 2-difluoromethylornithine (DFMO) (Cat#2761), ribavirin (Cat#R0077), and deoxyhypusine synthase inhibitor N1-guanyl-1,7-diaminoheptane (GC7) (Cat#259545) were purchased from Tocris, Tokyo Chemical Industry, and EMD Millipore respectively.

### Isolation of tonsillar B lymphocytes

Tonsillar tissues were acquired through the National Disease Research Interchange (NDRI, Philadelphia) and were collected from de-identified healthy donors via routine tonsillectomy procedures. Extraction of mononuclear cells from the tonsillar tissue was performed as previously described [87]. In brief, the block of tissue was cut into smaller (3mm) pieces, and cells were mechanically dissociated by using a stainless-steel sieve (250-μm mesh) with a syringe plunger, through which the tissue samples remained cold and moistened in the Hanks balanced salt solution (HBSS) with antibiotics (100 U/mL penicillin, 100 μg/mL streptomycin, 5 μg/mL gentamicin, 0.5 μg/mL Amphotericin B). Cell suspension was passed through a 40 μm plastic cell strainer and overlaid onto 10 mL Ficoll-Hypaque (GE Healthcare), followed by centrifugation (1000x g) for 20 mins at 4°C. Mononuclear cells were collected from the buffy coat at the interface, and washed three times with HBSS containing antibiotics and centrifuged (300x g) for 10mins at 4°C. Washed cells were resuspended in cryopreservation media (HyClone, Cat#SR30001.02) mixed with tonsil mononuclear cells complete media without antibiotics. B lymphocyte isolation was conducted through negative selection by using the B cell Isolation Kit II (Miltenyi Biotec, Cat#130-091-151) according to the manufacturer’s protocol. Purity of B lymphocytes (>98%) was assessed via immunostaining by using an anti-human-CD19, PE-conjugated antibody (Miltenyi Biotec, Cat#130-113-731) per manufacturer’s instructions and analyzed via flow cytometry on an Accuri C6 Plus (BD Biosciences).

### Preparation of KSHV viruses and de novo infection

iSLK.BAC16 cells were treated with 1μg/mL doxycycline and 1mM NaB for 24h, and then kept in fresh media only containing 1μg/mL doxycycline. Doxycycline was refreshed every 2 days. Supernatants were collected 6 dpi, centrifuged (400x g) for 10 mins to remove cellular debris, filtered through the 0.45μm filter and stored at -80°C. KSHV BAC16 viruses were titrated in HEK293T cells through the serial dilution of viral stock along with 8μg/mL polybrene via spinoculation (2500 rpm) for ~2h at 37°C. Percentage of GFP-positive cells was determined by flow cytometry on an Accuri C6 Plus. Forward Scatter (FS) and Side Scatter (SS) were used for gating of single cells. Analysis was performed using the FlowJo v10 software. Primary tonsillar B lymphocytes were spinoculated (2500 rpm) with KSHV BAC16 viruses (MOI = 3) along with 8μg/mL polybrene for ~2h at 37°C. Cell media was completely removed at 12hpi. Unbound viruses were washed away, and fresh media containing the tested compounds were added. Similarly, SLK and TIME cells were subjected to KSHV *de novo* infection (MOI = 0.5 and MOI = 1 respectively) with unbound viruses washed away and fresh media containing the tested compounds added 2h after viral spinoculation.

### Thin-layer chromatography

Protocol of thin-layer chromatography (TLC) to analyze polyamines was adapted from previous report [26,88,89]. Cells were harvested and resuspended in 2% perchloric acid solution (v/v) in ddH2O (Cat#SP339-500), sonicated at 4°C (30% amplitude, 1min total with 2sec ON and 2sec OFF), and incubated at 4°C for overnight on a rotating shaker. Cell lysates were centrifuged (11500x g) for 45mins at 4°C, and transferred to a new test tube. The following solutions were prepared freshly. 1 volume of cell lysates was mixed with 2 volumes of dansyl-chloride solution (18.6mM in Acetone; Cat#D-2625, Cat#BP392-100)) and 1 volume of supersaturated Sodium Carbonate solution (4.44M in ddH2O; Cat#S263-500), which was vortexed thoroughly and incubated for overnight in dark on a rotating shaker at room temperature. 0.5 volume of L-proline solution (1.3M in ddH2O; Cat#BP392-100) was added, then samples were thoroughly vortexed and incubated for another 1h. For extraction of dansylated polyamines, 2.5 volume of toluene (Cat# AC610951000) was added, and the mixtures were thoroughly vortexed and further incubated for 20 mins. The mixtures were centrifuged (13500 rpm) for 10mins at room temperature. The organic phase (upper layer) was carefully separated and spotted (~4uL) onto a TLC plate (Cat#1.05721.0001), and individual polyamines were separated by ascending chromatography using a cyclohexane:ethylacetate mixture (2,3) (v:v) as the eluant for 30 mins. Standards and controls were processed identically in parallel. The TLC plate was then imaged by using a FluorChem E (ProteinSimple) under UV light exposure.

### Cell transfection

Reverse transfection of siRNA was performed using Lipofectamine RNAiMAX (Invitrogen) as previously described [90]. Following Silencer Select siRNAs (Invitrogen) were used: ornithine decarboxylase 1 (ODC1) (s9821, s9822, s9823 as si1-3), agmatinase (AGMAT) (s379, s381 as si1-2), spermidine synthase (SMS) (s13173, s13175 as si1-2), spermine synthase (SRM) (s13430, s13432 as si1-2), deoxyhypusine synthase (DHPS) (s91, s92 as si1-2), and non-targeting control (Cat#AM4641). Turbofect (ThermoFisher) was used for vector transfection following manufacturer’s recommendations. Flag-tagged KSHV ORF50/RTA vector was a gift from Dr. Pinghui Feng (University of South California) [91], pSG5-BZLF1 was a gift from Dr. Diane Hayward (Addgene # 72637) [92], and pA3M-LANA was a gift from Dr. Erle Robertson (University of Pennsylvania). The pmaxGFP plasmid was obtained from Lonza.

### Immunoblotting

Protein immunoblotting was performed as previously described [93]. The following antibodies were used: anti-ODC1 (EMD Millipore, Cat#MABS36), anti-hypusine-eIF5A (EMD Millipore, Cat#ABS1064), anti-eIF5A (BD Biosciences, Cat#611976), anti-SSAT-1 (Novus, Cat#NB110-41622), anti-LC3I/II (CST, Cat#4108), anti-SQSTM1/p62 (D5E2) (CST, Cat#8025), anti-GAPDH (SCBT, Cat#sc-47724), anti-β-actin (SCBT, Cat#sc-47778), anti-DHPS (SCBT, Cat#sc-365077), anti-EBV-ZEBRA/Zta (SCBT, Cat#sc-53904), anti-HHV8-K8.1A/B (SCBT, Cat#sc-65446), anti-HHV8-ORF50/RTA (Abbiotec, Cat#251345), anti-KSHV-ORF26 (2F6B8) (Novus, Cat#NBP1-47357), anti-KSHV-LANA (Advanced Biotechnologies, cat# 13-210-100), anti-FLAG (CST, Cat#14793), anti-mouse-HRP (Invitrogen, Cat# 31430), anti-rabbit-HRP (Invitrogen, Cat#A27036), anti-rat-HRP (Invitrogen, Cat#A18739). An anti-KSHV-ORF45 antibody was a gift from Dr. Fanxiu Zhu (Florida State University).

### Dual-color immunostaining and flow cytometry

Cells were immune-stained as previously reported [93] by using anti-hypusine-eIF5A (EMD Millipore, Cat#ABS1064) and anti-eIF5A (BD Biosciences, Cat#611976) primary antibodies, and using fluorescently conjugated, anti-Mouse Alexa Fluor 488 and anti-Rabbit Alexa Fluor 647 secondary antibodies. Immune-stained cells were then analyzed by using an Accuri C6 Plus (BD Biosciences), and the data prepared by using the FlowJo v10 software.

### Real-Time quantitative PCR

The procedures of RNA extraction, cDNA preparation, genomic DNA extraction, and real-time quantitative PCR (RT-qPCR) assays were conducted as previously described [90]. Primers used in this study are listed in **S2 Table**.

### Analysis of RNAseq dataset

DESeq2 [94] and R package were utilized to analyze the differential gene expression of host genes in the publicly available datasets (GSE84237 [44] and GSE114625 [45]). Heatmap was generated by using PHeatmap in R (Kolde, R. Pheatmap version 1.0.12 (2019)), and hierarchical clustering was performed with the clustering_method = "complete". The color scale represents the z-score ($Z=\frac{x-\mu }{\sigma }$, with μ = mean and σ = standard deviation).

### Cell viability

CellTiter-Glo (Promega, Cat# G7570) was used for cell viability assays following the manufacturer’s instructions. Luminescence was measured on a BioTeK Cytation 5 plate reader.

### Protein sequence scanning

LANA and RTA protein sequences were retrieved from the NCBI database and scanned for hyp-eIF5A-dependency motifs [18] by using the ScanProsite webtool (https://prosite.expasy.org/prosite.html) [95]. Sequence of KSHV and human proteomes was retrieved from the UniProt database (respectively Proteome ID: UP000000942 and UP000005640). The bioinformatic program customized for this analysis is available at GitHub (https://github.com/ZhenyuWu-OSU/Motif_counting). Protein alignments were generated by using the BioEdit 7.2 software.

### Statistical analysis

Statistical analysis was performed by using GraphPad PRISM 5 or Excel. Results were presented as mean ± SEM. p-values were determined by using the two-tailed paired Student t-test, 1-way ANOVA with Tukey test as post hoc analysis and 2-way ANOVA with Bonferroni test as post hoc analysis.

## Supporting information

S1 Fig (A). A heatmap with hierarchical clustering was generated to illustrate the dysregulation of gene expression in polyamine pathway in BJAB cells latently infected with KSHV compared to non-infected BJAB cells (GEO accession: GSE114625). (B). Quantity of individual intracellular polyamine species (putrescine, spermidine, spermine) or total three species in TIME.BAC16 and naïve parental TIME cells at 8dpi was analyzed by TLC (top panel), and quantified relatively (right panel). Bottom panel showed the GFP fluorescence in these cells, indicating KSHV infection. (C, D). Intracellular polyamine species (putrescine, spermidine, spermine) in TREx BCBL1-RTA and BJAB cells treated with Dox for 48h or mock were analyzed by thin-layer chromatography (TLC) with pure individual polyamine species as reference (C). Quantity of individual polyamine species (Put, Spd, Spm) or total three species in TLC results was determined, and normalized to latency (D, left panel) or dox-induced lytic reactivation (D, right panel). Results were calculated from n = 2–4 independent experiments and presented as mean ± SEM (* p<0.05; *** p<0.001; 2-way ANOVA (Bonferroni test)).
(TIF)

S2 Fig (A). mRNA level of KSHV lytic genes (ORF50/RTA, K8) in un-induced HEK293.r219 cells transfected with ODC1 siRNAs (si1, si2, si3) or non-targeting control siRNA (NT) was analyzed by RT-qPCR assays and normalized to NT-transfected cells. (B, C). HEK293.r219 cells transfected with ODC1 siRNAs (si1, si2, si3) or NT were induced with ectopic expression of ORF50 for 48h and visualized by fluorescence imaging (B). mRNA level of KSHV lytic gene K8 in above cells was analyzed by RT-qPCR assays and normalized to NT-transfected ORF50-induced cells. (D, E). mRNA level of ODC1 (D) and KSHV lytic genes (ORF50/RTA, K8, ORF26; E) in iSLK.BAC16 cells transfected with ODC1 siRNAs (si1, si2, si3) or NT and subsequently induced with Dox (1μg/mL) + NaB (1mM) for 48h was analyzed by RT-qPCR assays and normalized to NT-transfected induced cells. Results were calculated from n = 3 independent experiments and presented as mean ± SEM (** p<0.01; *** p<0.001;, 1-way ANOVA (Tukey test) and 2-way ANOVA (Bonferroni test)).
(TIF)

S3 Fig (A, B). mRNA level of KSHV lytic genes (ORF50/RTA, K8) in un-induced HEK293.r219 cells transfected with siRNAs targeting SRM (si1, si2; A) or SMS (si1, si2; B), or NT was analyzed by qRT-assays and normalized to NT-transfected cells. (C, D). Protein level of SSAT-1 in iSLK.BAC16 cells treated with increasing doses of ribavirin for 24h was analyzed by immunoblotting assays (C). GAPDH was used as the loading control. Cell viability of above cells was analyzed by CellTiter-Glo assays and normalized to mock-treated cells (D). Results were calculated from n = 2–3 independent experiments and presented as mean ± SEM (* p<0.05; ** p<0.01; *** p<0.001; 1-way ANOVA (Tukey test) and 2-way ANOVA (Bonferroni test)).
(TIF)

S4 Fig (A). Cell viability of HEK293.r219 cells treated with or without exogenous polyamines (mixed polyamines supplement [PA Supp, 5x], Spermidine [Spd, 10μM] or Spermine [Spm, 10μM]) in the presence or absence of DFMO (500μM) for 24h was analyzed by CellTiter-Glo assays and normalized to mock-treated cells. (B). Protein level of KSHV lytic genes (K8.1A/B, ORF26) in TREx BCBL1-RTA cells pre-treated with DFMO (500μM) for 24h and subsequently induced with Dox (1μg/mL) for 48h was analyzed by immunoblotting assays. GAPDH was used as the loading control. (C). Cell viability of iSLK.BAC16 cells treated with increasing doses of clofazimine (CLF) for 24h was analyzed by CellTiter-Glo assays and normalized to mock-treated cells. (D). mRNA level of KSHV lytic genes (ORF50/RTA, K8/K-bZIP and ORF26) in iSLK.BAC16 cells pre-treated with the increasing doses of clofazimine (CLF) for 24h and subsequently induced by Dox (1μg/mL) for 48h were analyzed by RT-qPCR assays and normalized to mock-treated, induced cells. (E). Cell viability of TIME cells treated with the increasing doses of DFMO for 24h was analyzed by CellTiter-Glo assays and normalized to mock-treated cells. Results were calculated from n = 3 independent experiments and presented as mean ± SEM, (* p<0.05; ** p<0.01; *** p<0.001; 1-way ANOVA (Tukey test) and 2-way ANOVA (Bonferroni test)).
(TIF)

S5 Fig (A). Protein level of total eIF5A and hyp-eIF5A in KSHV latently infected iSLK.BAC16 cells and the parental SLK cells was analyzed by protein immunoblotting. (B). Protein level of DHPS, total eIF5A, and hyp-eIF5A in KSHV latently infected TIME.BAC16 cells at 8dpi was measured by protein immunoblotting and compared to uninfected controls. (C). Protein level of total eIF5A and hyp-eIF5A in iSLK.BAC16 cells pre-treated with DFMO (500μM) for 24 h and subsequently induced with Dox (1μg/mL) for 24h was analyzed by protein immunoblotting. GAPDH was used as the loading control. (D). Protein level of total eIF5A and hyp-eIF5A in HEK293.r219 cells induced with TPA (20ng/mL) + NaB (0.3mM) for 24h was analyzed by protein immunoblotting. Intensity of protein bands was determined by using the AlphaView SA software and normalized to GAPDH that was used as the loading control. Results were presented as mean ± SEM from n = 3–4 independent experiments (* p<0.05, two-tailed paired Student t-test).
(TIF)

S6 Fig (A). List of hyp-eIF5A-dependency motifs reported in Schuller et al. 2017 [18]. (B). Cell viability of iSLK.BAC16 cells treated with increasing doses of GC7 for 24h was analyzed by CellTiter-Glo assays and normalized to mock-treated cells. (C, D). SLK cells transfected with pmaxGFP (Lonza) and subsequently treated with GC7 (12.5μM) were visualized by fluorescence imaging (C). These cells were also analyzed by flow cytometry, and relative MFI of GFP expression as well as percentage of GFP-positive cells was determined (D). (E). List of hyp-eIF5A-dependency motifs in the coding sequence of EBV Zta protein [YP_401673.1]. (F, G). Sequence alignment of KSHV RTA (F) and LANA (G) proteins from different viral strains with the position of hyp-eIF5A-dependency motifs indicated in pink. Results were calculated from n = 3 independent experiments and presented as mean ± SEM (ns: not significant, two-tailed paired Student t-test).
(TIF)

S7 Fig (A, B). Cell viability of TREx BCBL1-RTA (A) and HEK293.r219 (B) cells treated with increasing doses of GC7 for 24h was analyzed by CellTiter-Glo assays and normalized to mock-treated cells. (C). mRNA level of indicated EBV lytic genes in Akata/BX cells pre-treated with GC7 for 24h and subsequently induced with human IgG for 48h was analyzed by RT-qPCR assays and normalized to mock-treated IgG-induced cells. (D, E). SLK cells were *de novo* infected with KSHV BAC16 viruses (MOI = 0.5). Unbound viruses were washed away, and cells were subsequently treated with the increasing doses of GC7 for 48h, then subjected to fluorescence imaging analysis (D). Nuclei were labelled with Hoechst (Blue), while GFP expression indicated infection with KSHV BAC16 viruses. MFI of GFP expression from five different fields of view for ≥7,000 cells was measured and normalized to mock-treated infected cells (E). (F). Cell viability of TIME cells treated with the increasing doses of GC7 for 24h was analyzed by CellTiter-Glo assays and normalized to mock-treated cells. (G). Relative mRNA level of KSHV lytic genes (ORF50/RTA, K8/K-bZIP) and latent gene (ORF73/LANA) upon primary B cells infection with KSHV BAC16 was analyzed by qPCR assays in comparison to mock (noise). Results were calculated from n = 2–3 independent experiments and presented as mean ± SEM.
(TIF)

S1 Table Analysis of hyp-eIF5A-dependency motifs existing in the HHV 8 type P (isolate GK18) proteome (Proteome ID: UP000000942). (XLSX)

S2 Table List of PCR primers used in this study. (XLSX)
