# Correction: Polyamine biosynthesis and eIF5A hypusination are modulated by the DNA tumor virus KSHV and promote KSHV viral infection

**DOI:** 10.1371/journal.ppat.1013496

**Published:** 2025-09-11

**Authors:** Guillaume N. Fiches, Zhenyu Wu, Dawei Zhou, Ayan Biswas, Tai-Wei Li, Weili Kong, Maxime Jean, Netty G. Santoso, Jian Zhu

The eighth author, Netty G. Santoso, should be noted as co-corresponding author. Netty G. Santoso’s email address is: Netty.Santoso@osumc.edu.
