# Supplementary figures and images for: Polyamine biosynthesis and eIF5A hypusination are modulated by the DNA tumor virus KSHV and promote KSHV viral infection
Source: PLoS Pathog. 2022 Apr 29;18(4):e1010503. doi: 10.1371/journal.ppat.1010503 (PMC9094511; doi:10.1371/journal.ppat.1010503)

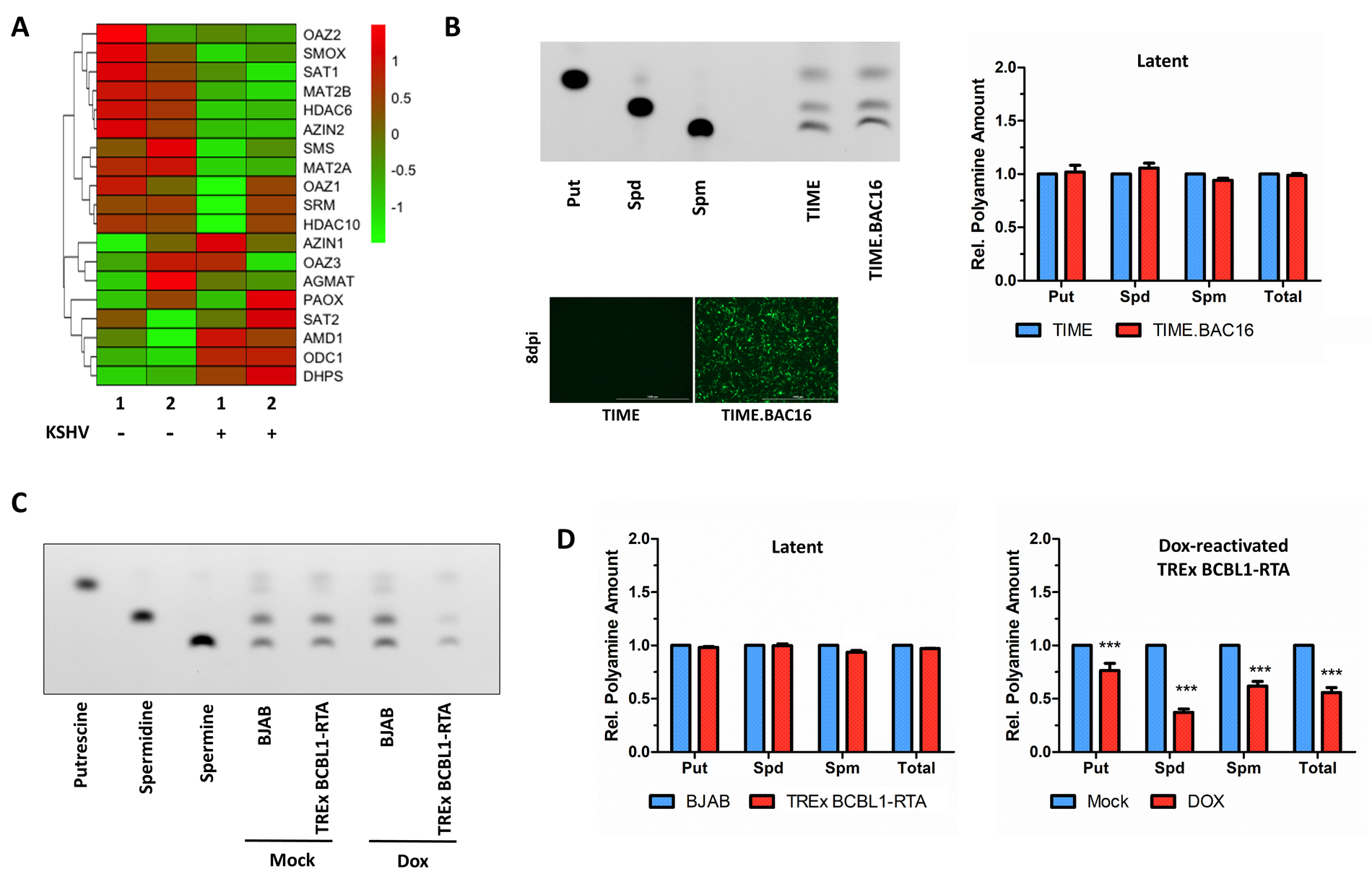

Supplement: S1 Fig — (A). A heatmap with hierarchical clustering was generated to illustrate the dysregulation of gene expression in polyamine pathway in BJAB cells latently infected with KSHV compared to non-infected BJAB cells (GEO accession: GSE114625). (B). Quantity of individual intracellular polyamine species (putrescine, spermidine, spermine) or total three species in TIME.BAC16 and naïve parental TIME cells at 8dpi was analyzed by TLC (top panel), and quantified relatively (right panel). Bottom panel showed the GFP fluorescence in these cells, indicating KSHV infection. (C, D). Intracellular polyamine species (putrescine, spermidine, spermine) in TREx BCBL1-RTA and BJAB cells treated with Dox for 48h or mock were analyzed by thin-layer chromatography (TLC) with pure individual polyamine species as reference (C). Quantity of individual polyamine species (Put, Spd, Spm) or total three species in TLC results was determined, and normalized to latency (D, left panel) or dox-induced lytic reactivation (D, right panel). Results were calculated from n = 2–4 independent experiments and presented as mean ± SEM (* p<0.05; *** p<0.001; 2-way ANOVA (Bonferroni test)). (TIF) [file ppat.1010503.s001.tif]

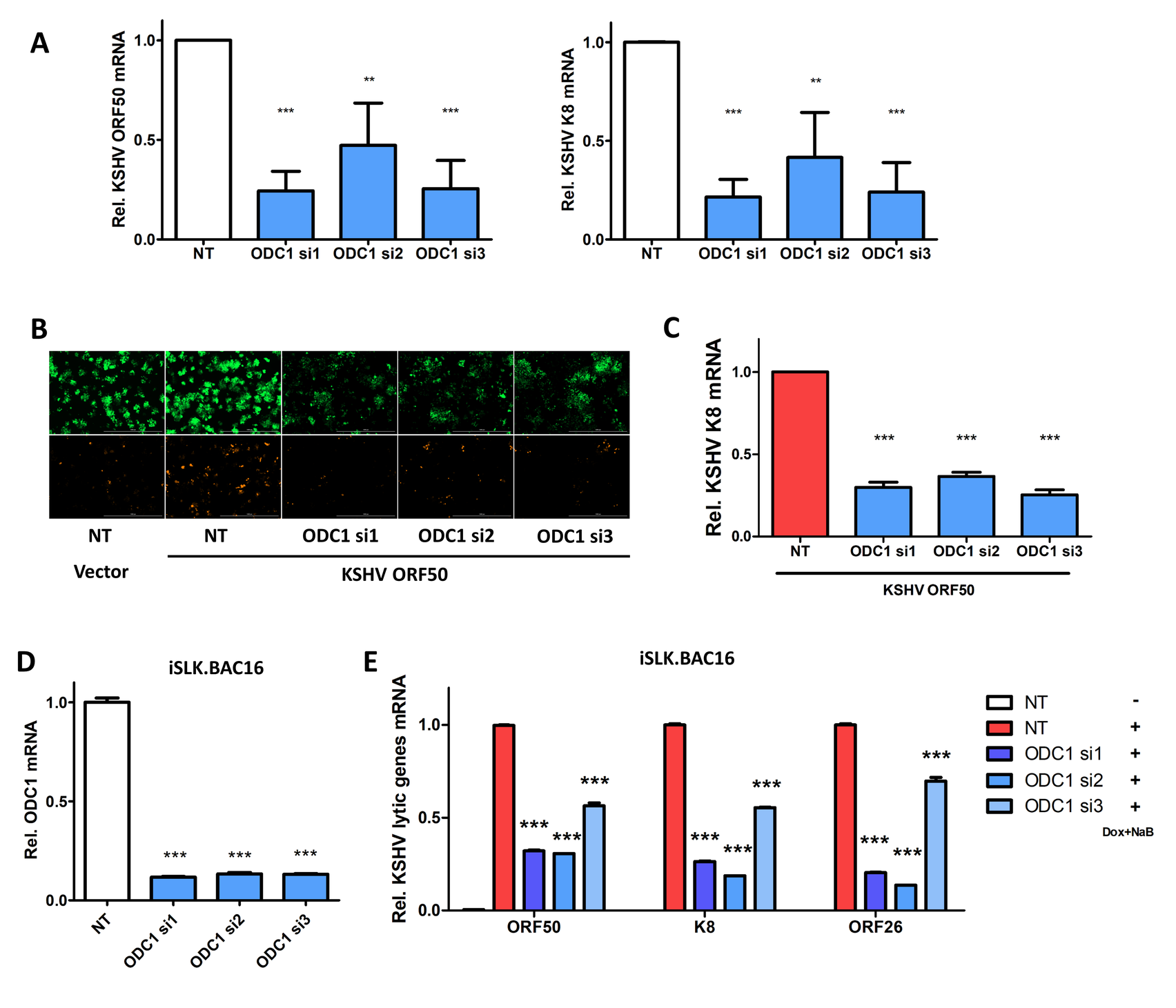

Supplement: S2 Fig — (A). mRNA level of KSHV lytic genes (ORF50/RTA, K8) in un-induced HEK293.r219 cells transfected with ODC1 siRNAs (si1, si2, si3) or non-targeting control siRNA (NT) was analyzed by RT-qPCR assays and normalized to NT-transfected cells. (B, C). HEK293.r219 cells transfected with ODC1 siRNAs (si1, si2, si3) or NT were induced with ectopic expression of ORF50 for 48h and visualized by fluorescence imaging (B). mRNA level of KSHV lytic gene K8 in above cells was analyzed by RT-qPCR assays and normalized to NT-transfected ORF50-induced cells. (D, E). mRNA level of ODC1 (D) and KSHV lytic genes (ORF50/RTA, K8, ORF26; E) in iSLK.BAC16 cells transfected with ODC1 siRNAs (si1, si2, si3) or NT and subsequently induced with Dox (1μg/mL) + NaB (1mM) for 48h was analyzed by RT-qPCR assays and normalized to NT-transfected induced cells. Results were calculated from n = 3 independent experiments and presented as mean ± SEM (** p<0.01; *** p<0.001;, 1-way ANOVA (Tukey test) and 2-way ANOVA (Bonferroni test)). (TIF) [file ppat.1010503.s002.tif]

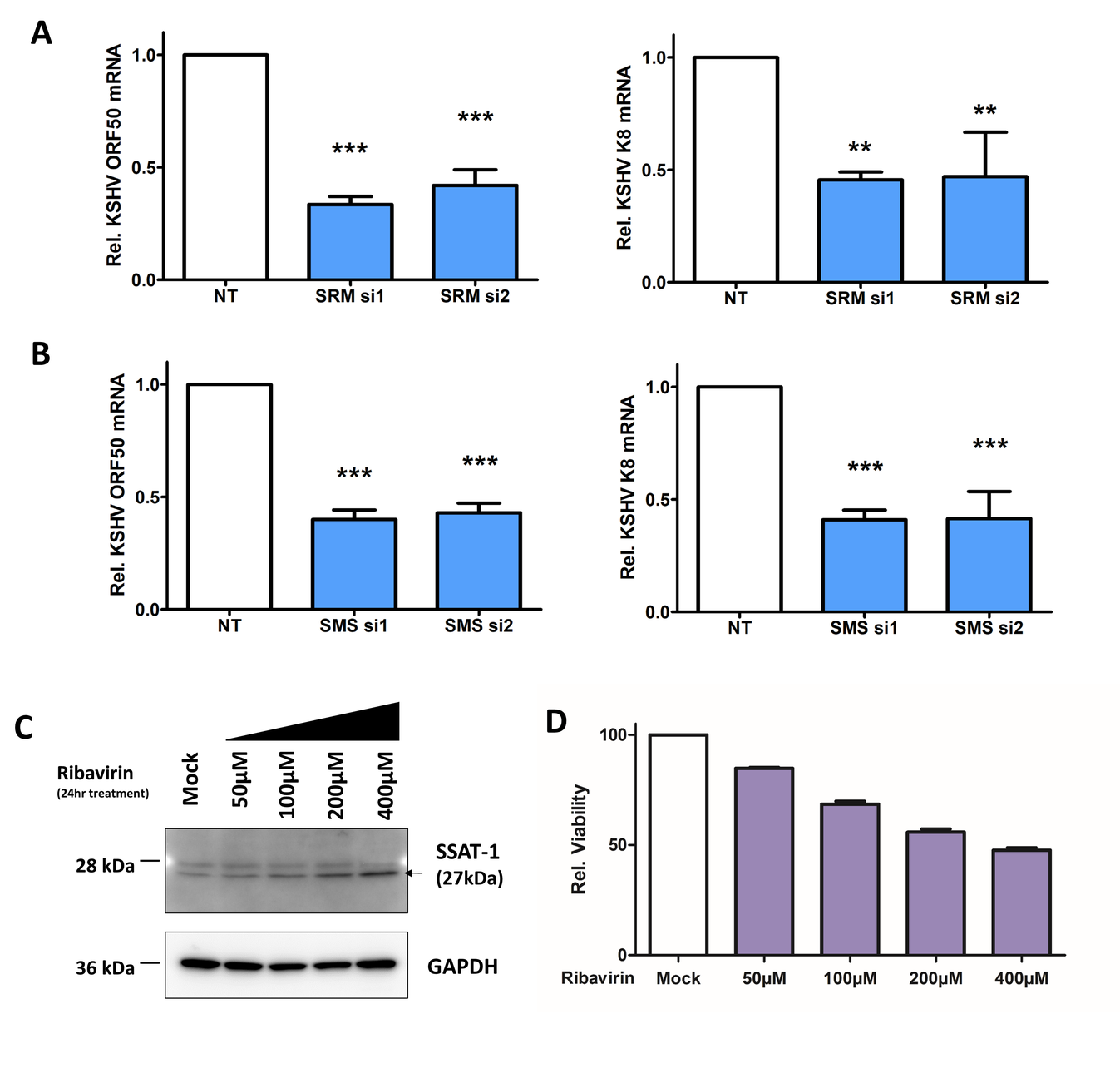

Supplement: S3 Fig — (A, B). mRNA level of KSHV lytic genes (ORF50/RTA, K8) in un-induced HEK293.r219 cells transfected with siRNAs targeting SRM (si1, si2; A) or SMS (si1, si2; B), or NT was analyzed by qRT-assays and normalized to NT-transfected cells. (C, D). Protein level of SSAT-1 in iSLK.BAC16 cells treated with increasing doses of ribavirin for 24h was analyzed by immunoblotting assays (C). GAPDH was used as the loading control. Cell viability of above cells was analyzed by CellTiter-Glo assays and normalized to mock-treated cells (D). Results were calculated from n = 2–3 independent experiments and presented as mean ± SEM (* p<0.05; ** p<0.01; *** p<0.001; 1-way ANOVA (Tukey test) and 2-way ANOVA (Bonferroni test)). (TIF) [file ppat.1010503.s003.tif]

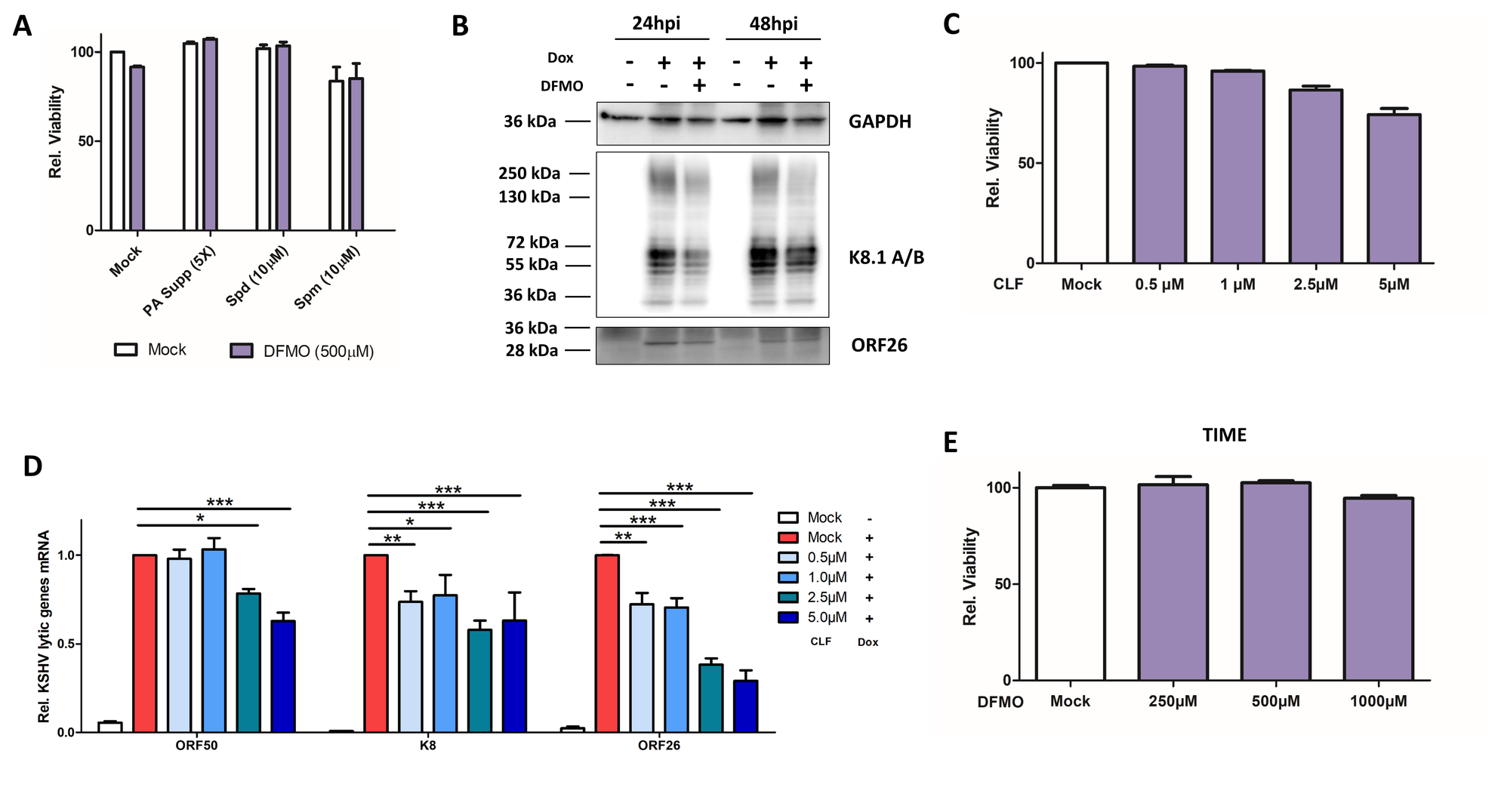

Supplement: S4 Fig — (A). Cell viability of HEK293.r219 cells treated with or without exogenous polyamines (mixed polyamines supplement [PA Supp, 5x], Spermidine [Spd, 10μM] or Spermine [Spm, 10μM]) in the presence or absence of DFMO (500μM) for 24h was analyzed by CellTiter-Glo assays and normalized to mock-treated cells. (B). Protein level of KSHV lytic genes (K8.1A/B, ORF26) in TREx BCBL1-RTA cells pre-treated with DFMO (500μM) for 24h and subsequently induced with Dox (1μg/mL) for 48h was analyzed by immunoblotting assays. GAPDH was used as the loading control. (C). Cell viability of iSLK.BAC16 cells treated with increasing doses of clofazimine (CLF) for 24h was analyzed by CellTiter-Glo assays and normalized to mock-treated cells. (D). mRNA level of KSHV lytic genes (ORF50/RTA, K8/K-bZIP and ORF26) in iSLK.BAC16 cells pre-treated with the increasing doses of clofazimine (CLF) for 24h and subsequently induced by Dox (1μg/mL) for 48h were analyzed by RT-qPCR assays and normalized to mock-treated, induced cells. (E). Cell viability of TIME cells treated with the increasing doses of DFMO for 24h was analyzed by CellTiter-Glo assays and normalized to mock-treated cells. Results were calculated from n = 3 independent experiments and presented as mean ± SEM, (* p<0.05; ** p<0.01; *** p<0.001; 1-way ANOVA (Tukey test) and 2-way ANOVA (Bonferroni test)). (TIF) [file ppat.1010503.s004.tif]

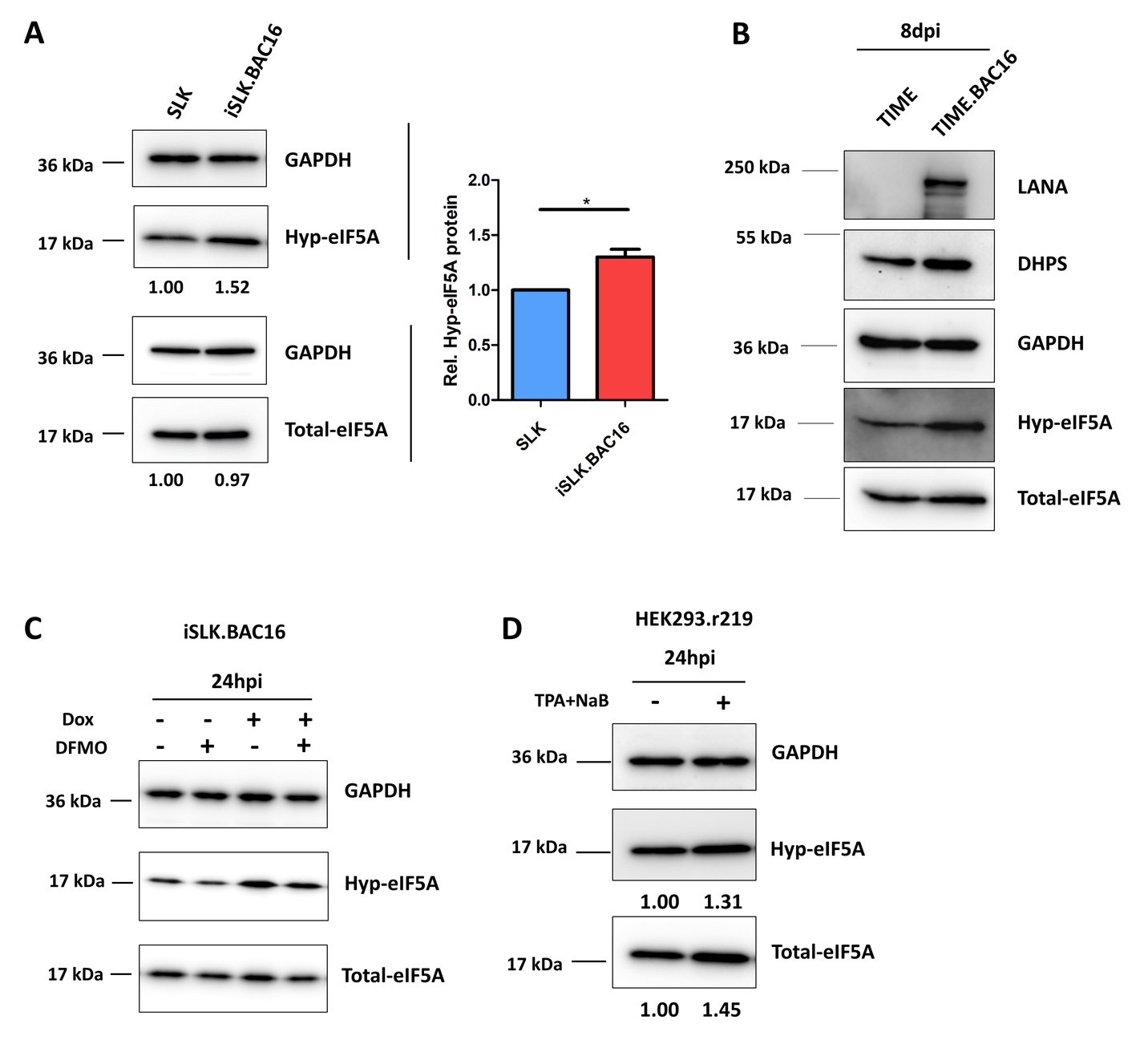

Supplement: S5 Fig — (A). Protein level of total eIF5A and hyp-eIF5A in KSHV latently infected iSLK.BAC16 cells and the parental SLK cells was analyzed by protein immunoblotting. (B). Protein level of DHPS, total eIF5A, and hyp-eIF5A in KSHV latently infected TIME.BAC16 cells at 8dpi was measured by protein immunoblotting and compared to uninfected controls. (C). Protein level of total eIF5A and hyp-eIF5A in iSLK.BAC16 cells pre-treated with DFMO (500μM) for 24 h and subsequently induced with Dox (1μg/mL) for 24h was analyzed by protein immunoblotting. GAPDH was used as the loading control. (D). Protein level of total eIF5A and hyp-eIF5A in HEK293.r219 cells induced with TPA (20ng/mL) + NaB (0.3mM) for 24h was analyzed by protein immunoblotting. Intensity of protein bands was determined by using the AlphaView SA software and normalized to GAPDH that was used as the loading control. Results were presented as mean ± SEM from n = 3–4 independent experiments (* p<0.05, two-tailed paired Student t-test). (TIF) [file ppat.1010503.s005.tif]

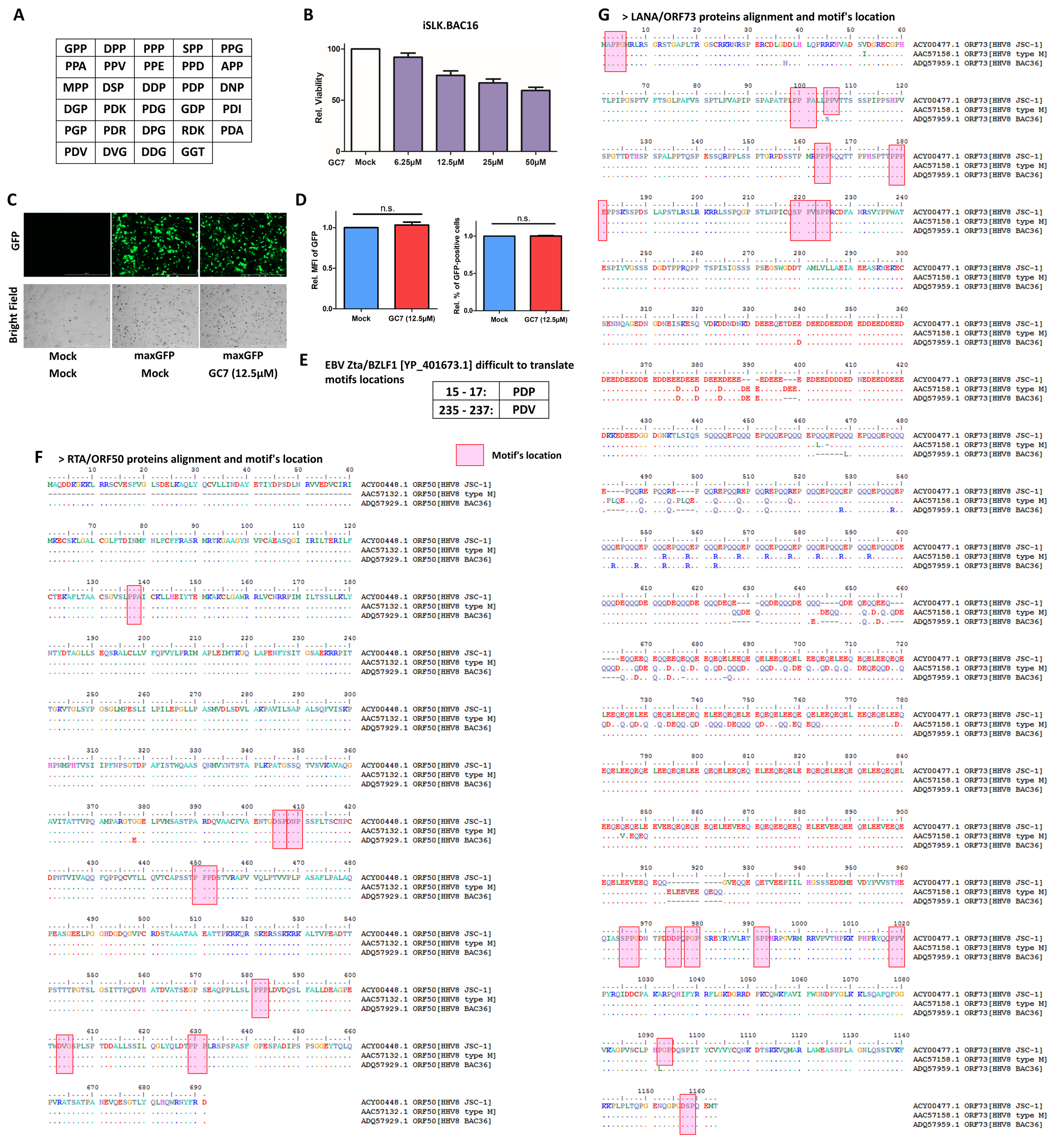

Supplement: S6 Fig — (A). List of hyp-eIF5A-dependency motifs reported in Schuller et al. 2017 [18]. (B). Cell viability of iSLK.BAC16 cells treated with increasing doses of GC7 for 24h was analyzed by CellTiter-Glo assays and normalized to mock-treated cells. (C, D). SLK cells transfected with pmaxGFP (Lonza) and subsequently treated with GC7 (12.5μM) were visualized by fluorescence imaging (C). These cells were also analyzed by flow cytometry, and relative MFI of GFP expression as well as percentage of GFP-positive cells was determined (D). (E). List of hyp-eIF5A-dependency motifs in the coding sequence of EBV Zta protein [YP_401673.1]. (F, G). Sequence alignment of KSHV RTA (F) and LANA (G) proteins from different viral strains with the position of hyp-eIF5A-dependency motifs indicated in pink. Results were calculated from n = 3 independent experiments and presented as mean ± SEM (ns: not significant, two-tailed paired Student t-test). (TIF) [file ppat.1010503.s006.tif]

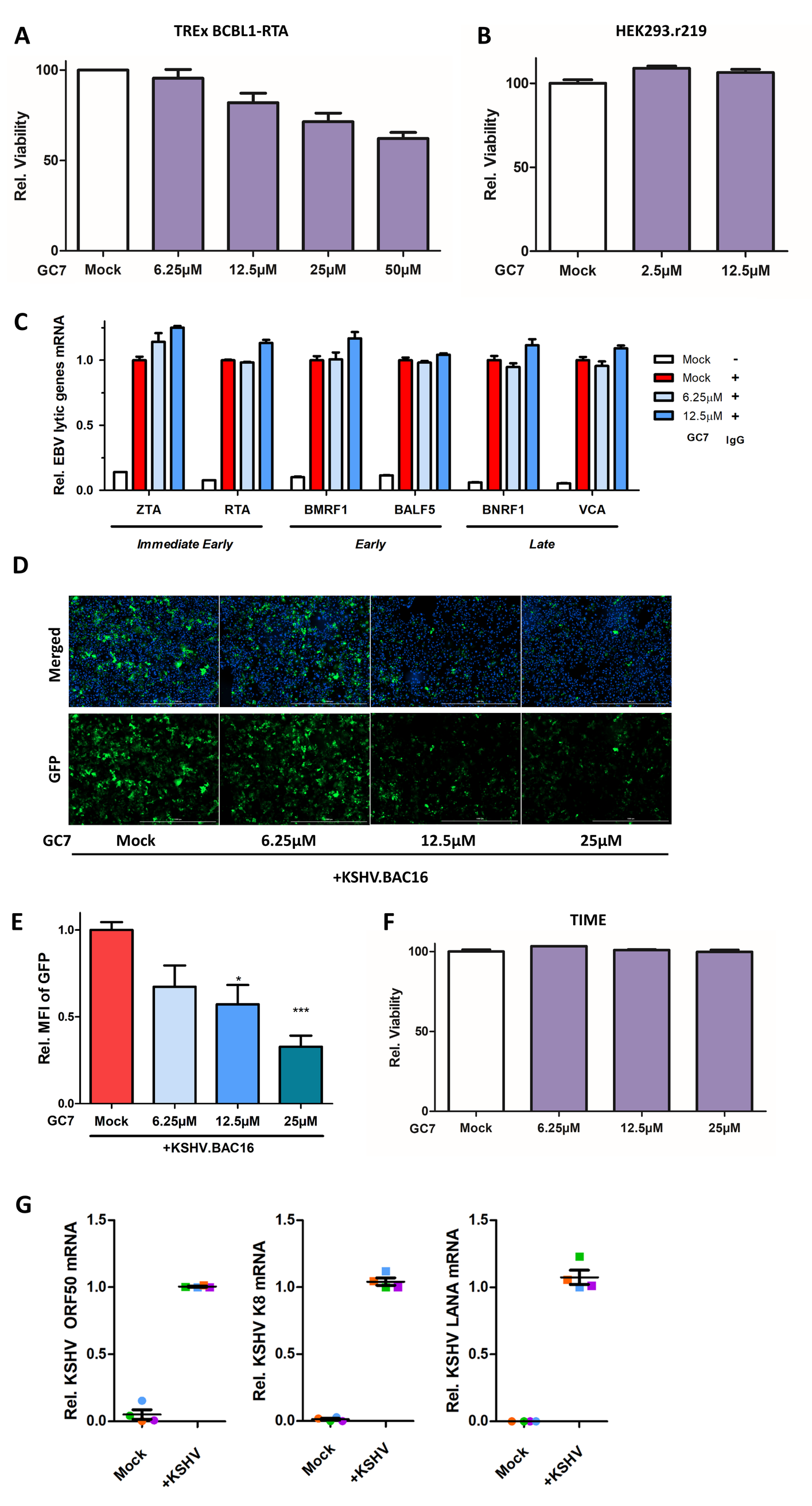

Supplement: S7 Fig — (A, B). Cell viability of TREx BCBL1-RTA (A) and HEK293.r219 (B) cells treated with increasing doses of GC7 for 24h was analyzed by CellTiter-Glo assays and normalized to mock-treated cells. (C). mRNA level of indicated EBV lytic genes in Akata/BX cells pre-treated with GC7 for 24h and subsequently induced with human IgG for 48h was analyzed by RT-qPCR assays and normalized to mock-treated IgG-induced cells. (D, E). SLK cells were de novo infected with KSHV BAC16 viruses (MOI = 0.5). Unbound viruses were washed away, and cells were subsequently treated with the increasing doses of GC7 for 48h, then subjected to fluorescence imaging analysis (D). Nuclei were labelled with Hoechst (Blue), while GFP expression indicated infection with KSHV BAC16 viruses. MFI of GFP expression from five different fields of view for ≥7,000 cells was measured and normalized to mock-treated infected cells (E). (F). Cell viability of TIME cells treated with the increasing doses of GC7 for 24h was analyzed by CellTiter-Glo assays and normalized to mock-treated cells. (G). Relative mRNA level of KSHV lytic genes (ORF50/RTA, K8/K-bZIP) and latent gene (ORF73/LANA) upon primary B cells infection with KSHV BAC16 was analyzed by qPCR assays in comparison to mock (noise). Results were calculated from n = 2–3 independent experiments and presented as mean ± SEM. (TIF) [file ppat.1010503.s007.tif]
